# Low-Density Lipoprotein Receptor (LDLR) Is Involved in Internalization of Lentiviral Particles Pseudotyped with SARS-CoV-2 Spike Protein in Ocular Cells

**DOI:** 10.3390/ijms241411860

**Published:** 2023-07-24

**Authors:** Sheetal Uppal, Olga Postnikova, Rafael Villasmil, Igor B. Rogozin, Alexander V. Bocharov, Thomas L. Eggerman, Eugenia Poliakov, T. Michael Redmond

**Affiliations:** 1 Laboratory of Retinal Cell & Molecular Biology, National Eye Institute, National Institutes of Health, Bethesda, MD 20892, USA; sheetal.uppal2@nih.gov (S.U.); olga.postnikova@nih.gov (O.P.); 2 Flow Cytometry Core Facility, National Eye Institute, National Institutes of Health, Bethesda, MD 20892, USA; villasmilr@nei.nih.gov; 3 National Center for Biotechnology Information, National Library of Medicine, National Institutes of Health, Bethesda, MD 20894, USA; rogozin@ncbi.nlm.nih.gov; 4 Clinical Center, National Institutes of Health, Bethesda, MD 20894, USA; abocharov@mail.cc.nih.gov (A.V.B.); thomas.eggerman@nih.gov (T.L.E.); 5 National Institute of Diabetes, Digestive and Kidney Diseases, National Institutes of Health, Bethesda, MD 20892, USA

**Keywords:** LDL receptor, low-density lipoprotein receptor, LDLR, SARS-CoV-2, spike protein, S-protein, ARPE-19, ocular cells

## Abstract

Here, we present evidence that caveolae-mediated endocytosis using LDLR is the pathway for SARS-CoV-2 virus internalization in the ocular cell line ARPE-19. Firstly, we found that, while Angiotensin-converting enzyme 2 (ACE2) is expressed in these cells, blocking ACE2 by antibody treatment did not prevent infection by SARS-CoV-2 spike pseudovirions, nor did antibody blockade of extracellular vimentin and other cholesterol-rich lipid raft proteins. Next, we implicated the role of cholesterol homeostasis in infection by showing that incubating cells with different cyclodextrins and oxysterol 25-hydroxycholesterol (25-HC) inhibits pseudovirion infection of ARPE-19. However, the effect of 25-HC is likely not via cholesterol biosynthesis, as incubation with lovastatin did not appreciably affect infection. Additionally, is it not likely to be an agonistic effect of 25-HC on LXR receptors, as the LXR agonist GW3965 had no significant effect on infection of ARPE-19 cells at up to 5 μM GW3965. We probed the role of endocytic pathways but determined that clathrin-dependent and flotillin-dependent rafts were not involved. Furthermore, 20 µM chlorpromazine, an inhibitor of clathrin-mediated endocytosis (CME), also had little effect. In contrast, anti-dynamin I/II antibodies blocked the entry of SARS-CoV-2 spike pseudovirions, as did dynasore, a noncompetitive inhibitor of dynamin GTPase activity. Additionally, anti-caveolin-1 antibodies significantly blocked spike pseudotyped lentiviral infection of ARPE-19. However, nystatin, a classic inhibitor of caveolae-dependent endocytosis, did not affect infection while indomethacin inhibited only at 10 µM at the 48 h time point. Finally, we found that anti-LDLR antibodies block pseudovirion infection to a similar degree as anti-caveolin-1 and anti-dynamin I/II antibodies, while transfection with LDLR-specific siRNA led to a decrease in spike pseudotyped lentiviral infection, compared to scrambled control siRNAs. Thus, we conclude that SARS-CoV-2 spike pseudovirion infection in ARPE-19 cells is a dynamin-dependent process that is primarily mediated by LDLR.

## 1. Introduction

Beginning with the sequencing of the novel coronavirus SARS-CoV-2 RNA genome, the scientific community has moved quickly in the study of SARS-CoV-2, resolving the crystal structure of spike protein and developing various immunoassays for SARS-CoV-2 spike protein [1,2]. The ACE2 receptor was identified as the receptor for SARS-CoV-2 spike protein in HeLa cells [2], and these results were extended and confirmed by structural studies [3,4]. It was concluded that SARS-CoV-2 uses the ACE2 receptor and the cellular serine protease TMPRSS2 for S protein priming in its internalization process, analogous to the SARS virus [5].

Despite this, there has been accumulating information that various tissues and cells with low ACE2 expression can still be infected with the SARS-CoV-2 virus. For example, adipose tissues and ocular sclera are infected independently of the ACE2 receptor [6,7]. In addition, it was demonstrated that SARS-CoV-2 may infect T-lymphocytes and epithelial cells independently of ACE2 [8,9]. In this regard, CD4 and LFA-1 receptors were proposed to interact with spike protein and mediate infection of T-lymphocytes [9,10,11]. In addition, it was proposed that SARS-CoV-2 cellular entry into Baby Hamster Kidney fibroblasts overexpressing the human ACE2 receptor (BHK-ACE2) is independent of the ACE2 cytoplasmic domain signaling and requires additional co-factors or co-receptors to be internalized into cells [12]. The observed incomplete inhibition mediated by ACE2 antibodies and the high neutralization potency of monoclonal antibodies (mAbs) targeting the N-terminal domain of SARS-CoV-2-S1 and not the receptor-binding domain region suggest that SARS-CoV-2 might use other mechanisms for host cell entry [13]. Furthermore, the latest evidence demonstrates that SARS-CoV-2 entry in different cells is promoted by additional receptors, including AXL, Neuropilin-1 (NRP-1), Basigin (CD147), AT1 (Angiotensin II receptor type 1), and AVPR1B (Vasopressin V1b receptor) proteins [14,15,16,17]. The latest studies on newly arising SARS-CoV-2 variants have placed a higher emphasis on various endocytic pathways [18]. Thus, coronavirus infection may employ distinct endocytic pathways, for example, clathrin-dependent and caveolae-dependent pathways [19,20]. Some groups have proposed targeting the endocytic pathway and lipid raft microdomains to block SARS-CoV-2 infection [21]. Several other candidate receptors, such as EGFR, AXL, and LDL receptor (LDLR), have been predicted by computational screening [16], and several alternative receptors, such as CD147, AXL, and SRB1, were considered from additional experimental data [15,16].

Besides respiratory surface cells, the major portal of entry for the SARS-CoV-2 virus, the eyes may be responsible for a fraction of SARS-CoV-2 infections, though this remains a matter of debate [22,23]. Anecdotally, the recent COVID variant X.B.B. 1.16 (“Arcturus”) may cause conjunctivitis in children [24]. However, ocular surface cells, in particular cells of the limbus and sclera, expressing ACE2 and TMPRSS2, are capable of being infected by the SARS-CoV-2 virus [6,7]. How important these receptors are in the infection of the limbus and scleral cells or other ocular cells is central to resolving the role of ocular cells in SARS-CoV-2 infection.

Here, we demonstrate that lentiviral pseudovirions pseudotyped with SARS-CoV-2 spike protein can infect ARPE-19, a human retinal pigment epithelium cell line, in culture, and that this infection could be blocked by anti-LDLR, anti-Caveolin1, and anti-Dynamin antibodies, cholesterol-depletion agents, as well as by LDLR siRNAs. We propose that caveolae and LDLR receptors in caveolae are components of the receptor-dependent endocytosis machinery that the SARS-CoV-2 virus uses to infect certain tissues, including ocular cells.

## 2. Results

### 2.1. Spike Pseudovirions Can Infect ARPE-19 Independently of ACE2

**Figure 1:**
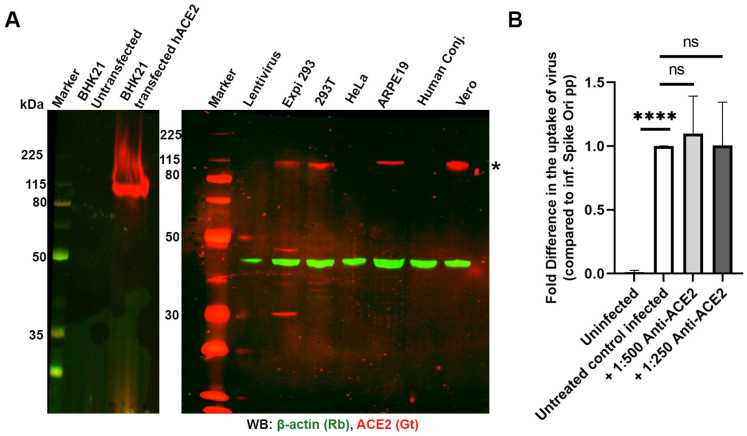
(**A**) Expression of ACE2 in different cell lines probed with anti-ACE2 antibodies (AF933, 1:1000 dilution, 0.5 µg/mL). Expression of ACE2 (*; red) in BHK21 cells untransfected or transfected with human ACE2 construct (positive control, left panel) and various other cell lines showing expression in ARPE-19 cells (right panel). (**B**) Blocking ACE2 does not affect pseudovirion uptake at MOI 10 in ARPE-19 cells. Fold difference in uptake of pseudovirions in the presence of 1:500 dilution (1 µg/mL) and 1:250 dilution (2 µg/mL) of AF933 ACE2 antibody after 24 h incubation. **** *p* < 0.0001, ns—*p* > 0.05.

Numerous sources have demonstrated that the SARS-CoV-2 spike protein utilizes ACE2 as the receptor for the majority of cellular infections. We were interested in determining the point of entry in the RPE cell culture model, ARPE-19. First, we demonstrated that ACE2 is present in ARPE-19 cells using two different antibodies (AF933 and MAB933, which were tested on unmodified BHK cells and ACE2-overexpressing BHK cells; Figure 1A, Supplementary Figure S1A). Second, we utilized these two antibodies in dilutions ranging from 1:50 to 1:500 to block spike-displaying lentiviral pseudovirions but did not observe any significant inhibition of infection of ARPE-19 cells (Figure 1B and Supplementary Figure S1B).

**Figure 2:**
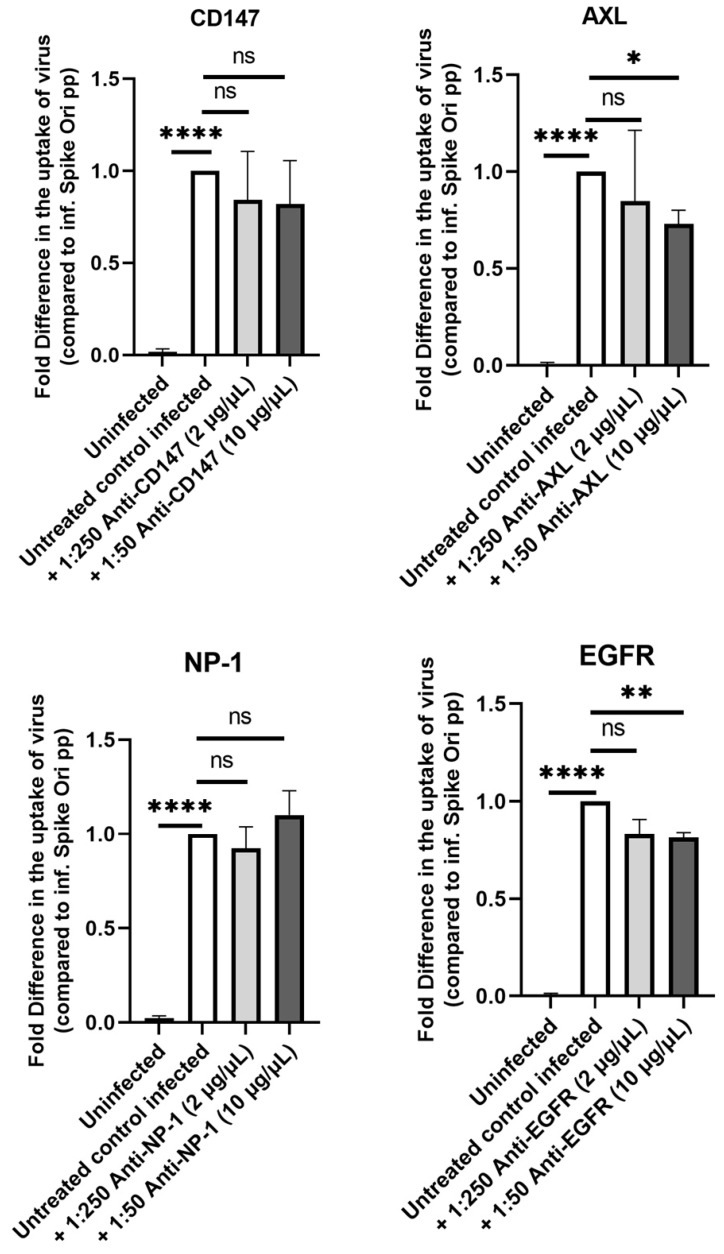
Lack of effect on pseudovirion uptake by blocking several alternate S protein receptors. Fold difference in uptake of spike-pseudotyped lentiviruses at MOI 10 in the presence of various dilutions of anti-CD147 (# 306221) (**top left panel**), anti-Neurophilin-1 (# MAB3870) (**bottom left panel**), anti-AXL (PA5-34658) (**top right panel**) and anti-EGFR (05-104) (**bottom right panel**) antibodies after 24 h incubation. Inf. Spike ori pp, infection with Spike protein original pseudotyped particles. * *p* <  0.01, ** *p*  <  0.001, **** *p* < 0.0001, ns—*p* > 0.05.

Next, we investigated whether cholesterol-rich lipid rafts could be implicated in the SARS-CoV-2 infection of ARPE-19 cells, as has been found for some other cells [25,26]. Extracellular vimentin was shown to expedite SARS-CoV-2 entry through the ACE2 receptor in human endothelial cells, and antibodies against vimentin prevented interaction with the SARS-CoV-2 spike protein and inhibited SARS-CoV-2 entry [27]. We tested antibodies against vimentin (Supplemental Figure S2) and other proposed candidate receptors for the SARS-CoV-2 virus—Neuropilin-1, EGFR, AXL, and CD147—but did not detect significant effects on ARPE-19 infection [15,16,28] (Figure 2).

### 2.2. Perturbation of Cholesterol Homeostasis Inhibits Spike-Pseudovirion Infection of ARPE-19

#### 2.2.1. Cyclodextrins

**Figure 3:**
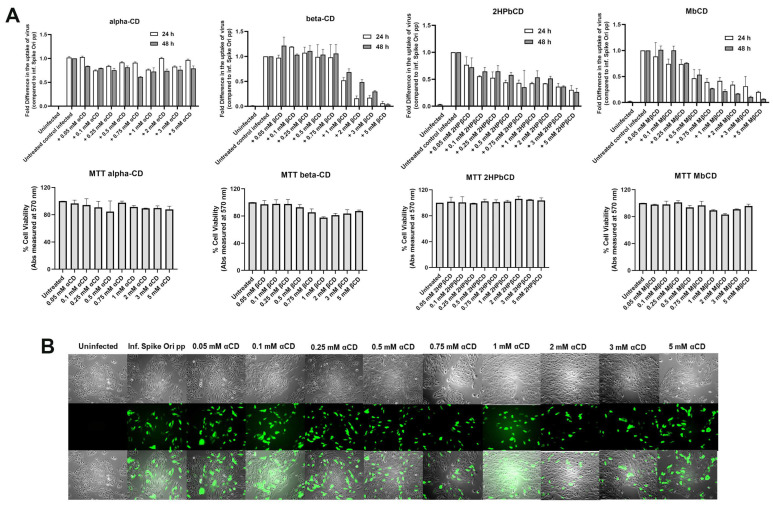
(**A**) Various β-cyclodextrins inhibit S protein pseudovirion uptake in ARPE-19 cells. Fold difference in uptake of original spike-pseudotyped lentiviruses at MOI 10 in the presence of 0.05–5 mM concentrations of cyclodextrins α-CD, β-CD, 2-HPβ-CD and Mβ-CD (top set of panels, from left to right respectively) at 24 h (□) and 48 h (■). (**B**) GFP fluorescence (green) analysis of infected ARPE-19 cells with Expi293-produced pseudovirions in the presence of various concentrations of α-CD; α-CD, α-cyclodextrin; β-CD, β-cyclodextrin; 2-HPβ-CD, 2-Hydroxypropyl-β-cyclodextrin; and Mβ-CD, methyl-β-cyclodextrin. 4× magnification, scale bar: 180 µm.

It was recently established that SARS-CoV-2 requires cholesterol for viral entry [29]. To perturb cholesterol homeostasis in ARPE-19 cells, we used cyclodextrins and oxysterols. Cyclodextrins are beneficial for decreasing cholesterol accumulation in patients [30,31,32]. It is established that α-cyclodextrin (α-CD) interacts with phospholipids in membranes, while various β-cyclodextrins (β-CDs) participate in cholesterol homeostasis and its direct removal from membranes [33,34]. Recently, it was proposed that the interaction of β-CDs with phospholipids leads to clinical benefits [35]. On the other hand, several groups have proposed that SARS-CoV-2 infection is dependent on cholesterol-rich lipid rafts, as was previously demonstrated for SARS [25,26]. We found that β-CD, methyl-β-cyclodextrin (Mβ-CD), and 2-hydroxypropyl-β-cyclodextrin (2HPβ-CD) inhibit infection by pseudovirions but that α-CD does not (Figure 3A, upper panel, and Figure 3B). None of the cyclodextrins affected the viability of cells (Figure 3A, lower panel).

2HPβ-CD was proposed to lower tissue cholesterol via multiple mechanisms, including those mediated by oxysterols [36]. Furthermore, oxysterols (25-hydroxycholesterol (25-HC) and 27-hydroxycholesterol) were found to sequester human rotavirus (HRV) particles into late endosomes and prevent the hijacking by HRV of the cholesterol recycling pathway between ER and late endosomes [37]. Additionally, we observed that Bafilomycin A1, a known endosomal/lysosomal acidification agent, effectively blocked pseudovirion infection of ARPE-19 cells (Supplemental Figure S3), as previously described for SARS-CoV-2 infection of mice [38].

#### 2.2.2. Oxysterol 25-HC

**Figure 4:**
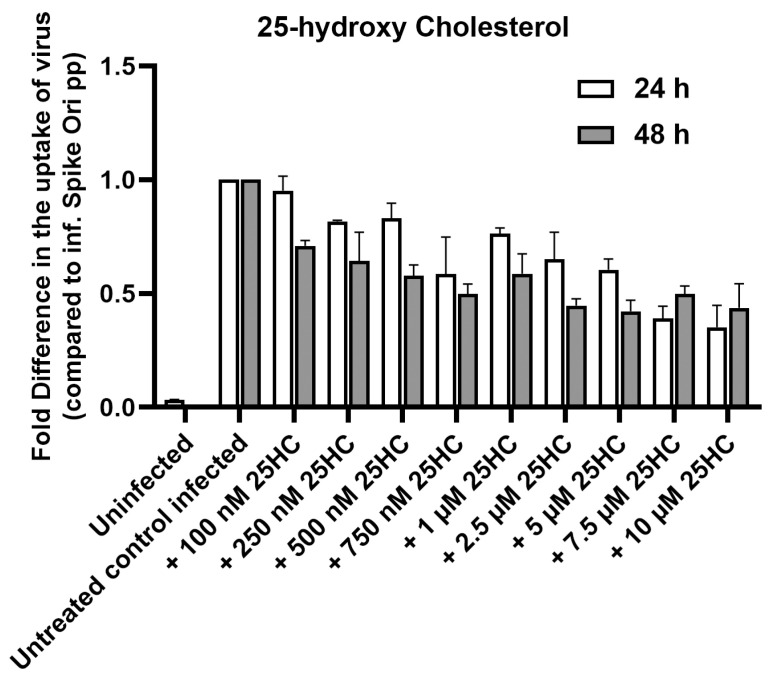
25-hydroxycholesterol (25-HC) inhibits S protein pseudovirion infection of ARPE-19 cells in a dose-dependent manner Fold difference in uptake of original spike-pseudotyped lentiviruses at MOI 10 in the presence of 100 nM–10 µM concentrations of 25-HC at 24 h (□) and 48 h (■) compared to untreated controls infected with original spike protein pseudotyped particles.

The oxysterol 25-hydroxycholesterol is well known as an antiviral mediator and has antiviral activity in cell culture against a variety of enveloped and non-enveloped viruses [39,40,41,42,43]. The gene encoding cholesterol 25-hydroxylase that produces 25-HC was found to be one of the more strongly induced IFN-stimulated genes in a screen of several hundred genes [40,44]. 25-HC has also been shown to be a potent SARS-CoV-2 inhibitor [45]. Therefore, we incubated ARPE-19 cells treated with SARS-CoV-2 native spike pseudotyped lentiviral particles with 0–10 μM 25-HC. We found that 25-HC inhibits pseudovirion infection of ARPE-19 in a dose-dependent manner (Figure 4), so we decided to probe the mode of action of 25-HC.

**Figure 5:**
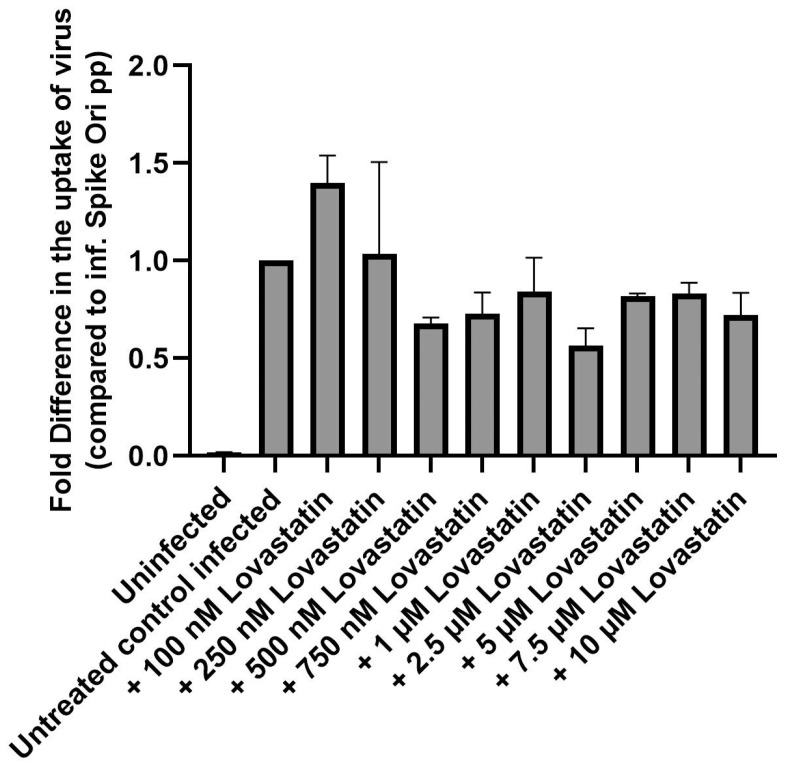
Lovastatin has little effect in blocking S protein pseudovirion infection of ARPE-19 cells. Fold difference in uptake of original spike-pseudotyped lentiviruses at MOI 10 in the presence of 100 nM–10 μM concentrations of lovastatin at 48 h compared to untreated controls infected with original spike protein pseudotyped particles alone.

It has been hypothesized that oxysterols might have a role in the negative regulation of sterol synthesis during viral infection. To determine if cholesterol biosynthesis is involved, we utilized lovastatin, which inhibits the rate-limiting step of cholesterol biosynthesis (HMG CoA reductase). We used a 6-h preincubation period with 0–10 μM lovastatin alone, followed by a 48-h period of co-incubation with lovastatin and pseudovirions. We found that lovastatin showed only a slight decrease in infection at all concentrations, so we conclude that cholesterol biosynthesis is not a major pathway in the antiviral effect of 25-HC (Figure 5).

**Figure 6:**
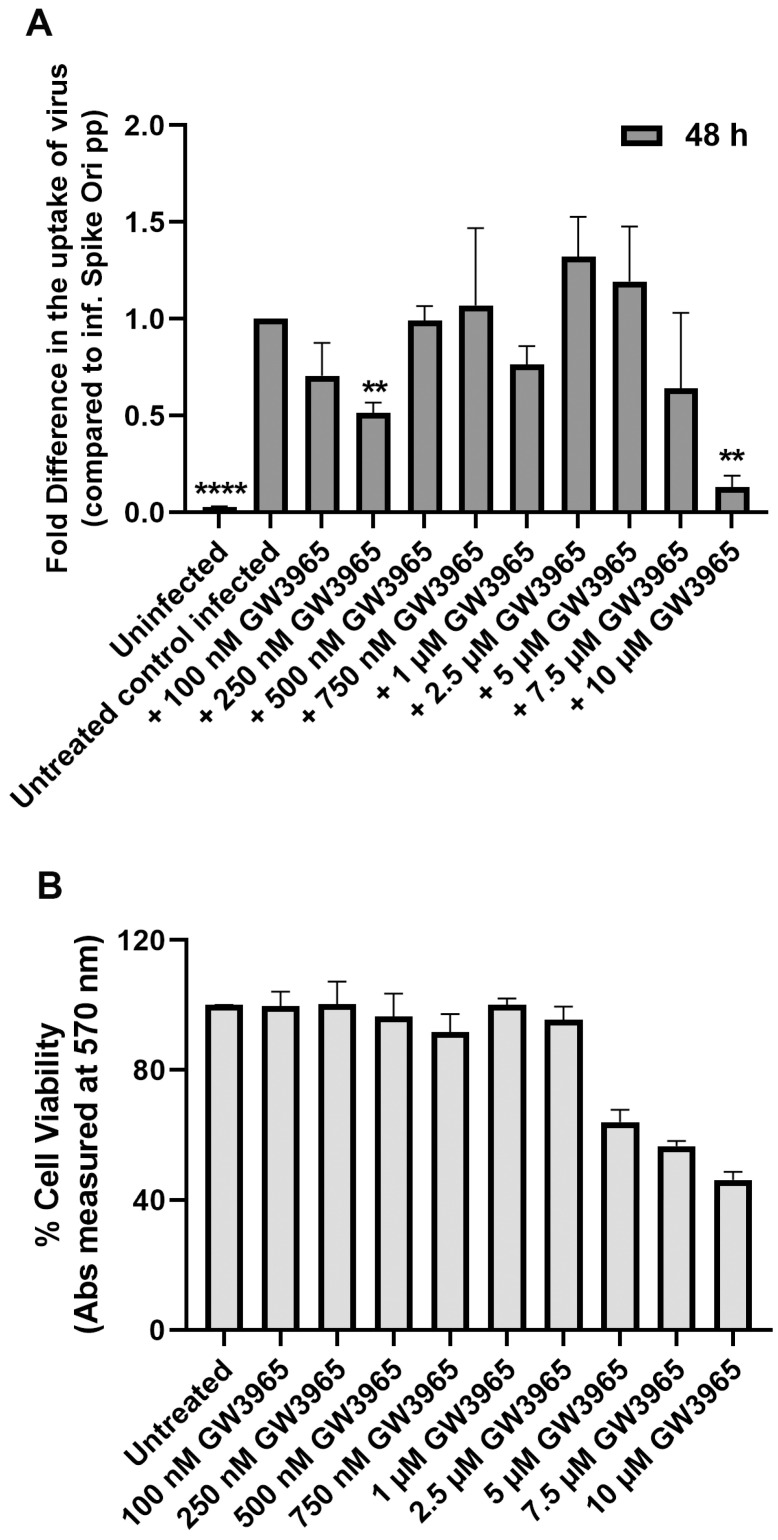
(**A**) LXR activation has little effect on blocking S protein pseudovirion infection of ARPE-19 cells. Fold difference in uptake of original spike-pseudotyped lentiviruses at MOI 10 in the presence of 100 nM–10 μM concentrations of the synthetic LXR agonist GW3965 at 48 h compared to untreated controls infected with original spike protein pseudotyped particles alone. (**B**) GW3965 is toxic at the highest 10 μM concentration: % Cell viability at 100 nM–10 μM concentrations of GW3965 measured by MTT assay. ** *p*  <  0.001, **** *p* < 0.0001.

Next, we asked if the agonistic effect of 25-HC on LXR receptors is the reason for its inhibition of viral infection [46,47]. LXR activation stimulates LDLR ubiquitination and degradation, limiting further uptake of exogenous cholesterol [46,47,48]. We preincubated cells with 0–10 μM of the synthetic LXR agonist GW3965 and observed no significant effect on spike-pseudotyped lentiviral infection of ARPE-19 cells up to 5 mM concentration (Figure 6A). We could not use higher concentrations because, at higher concentrations, we detected up to 50% cell death (Figure 6B).

### 2.3. Clathrin-Dependent and Flotillin-Dependent Endocytic Pathways Are Not Major Pathways in Internalization of SARS-CoV-2 Spike Pseudotyped Lentiviral Particles

**Figure 7:**
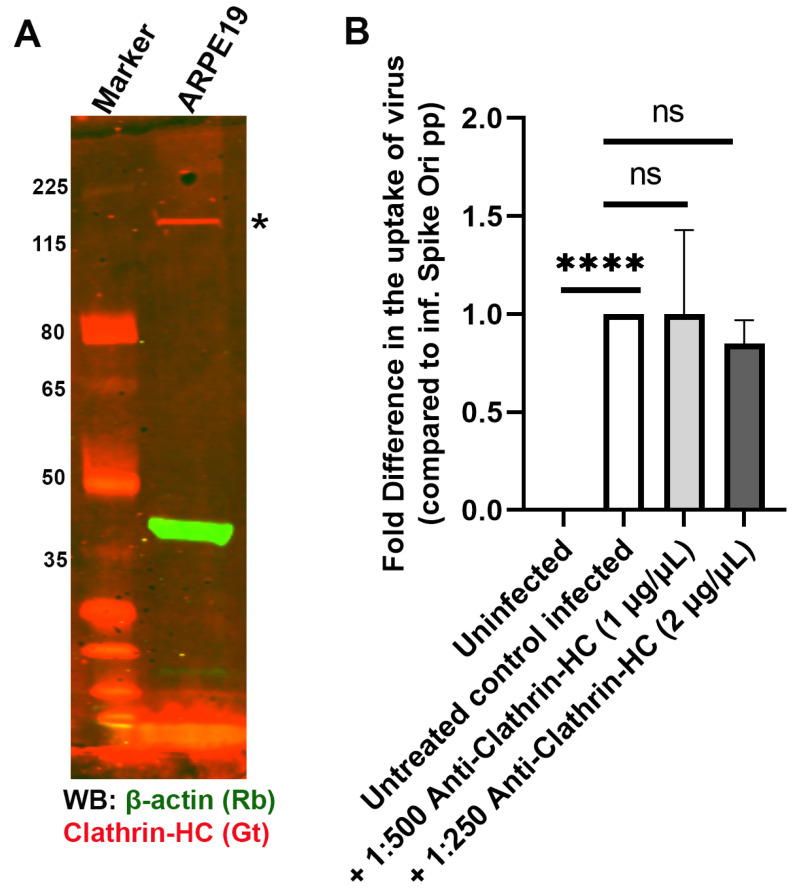
Antibody-mediated blocking of clathrin has no effect in blocking S protein pseudovirion infection of ARPE-19 cells. (**A**) Clathrin is expressed in ARPE-19 cells (*; red). Immunoblot of clathrin with anti-clathrin HC antibody (red) and β-actin (green) in ARPE-19 cells. (**B**) Fold difference in uptake of original spike-pseudotyped lentiviruses at MOI 10 in the presence of anti-clathrin antibody at 1:500 dilution (1 µg/mL) and 1:250 dilution (2 µg/mL) at 24 h compared to untreated controls infected with original spike protein pseudotyped particles alone. **** *p* < 0.0001, ns—*p* > 0.05.

To better understand the entry point of pseudovirions in ARPE-19 cells, we probed several endocytic pathways that could be involved in infection [49]. The SARS-CoV-2 spike protein undergoes rapid internalization via CME in an ACE2-dependent manner in several cell models of SARS-CoV-2 infection [50]. However, in other models, ACE2-dependent internalization of SARS-CoV-2 pseudovirions was clathrin and dynamin-independent [25]. We determined that clathrin-specific antibodies did not block entry of the SARS-CoV-2 spike pseudotyped lentivirus in ARPE19 (Figure 7).

**Figure 8:**
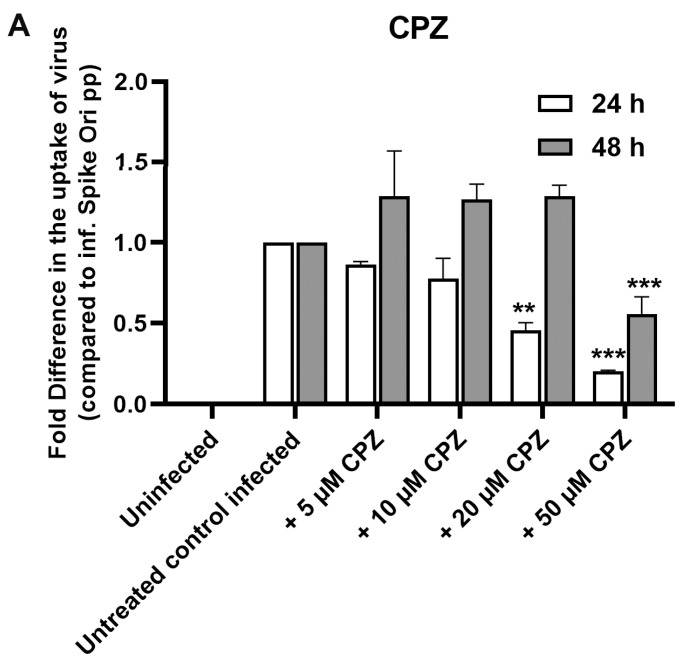

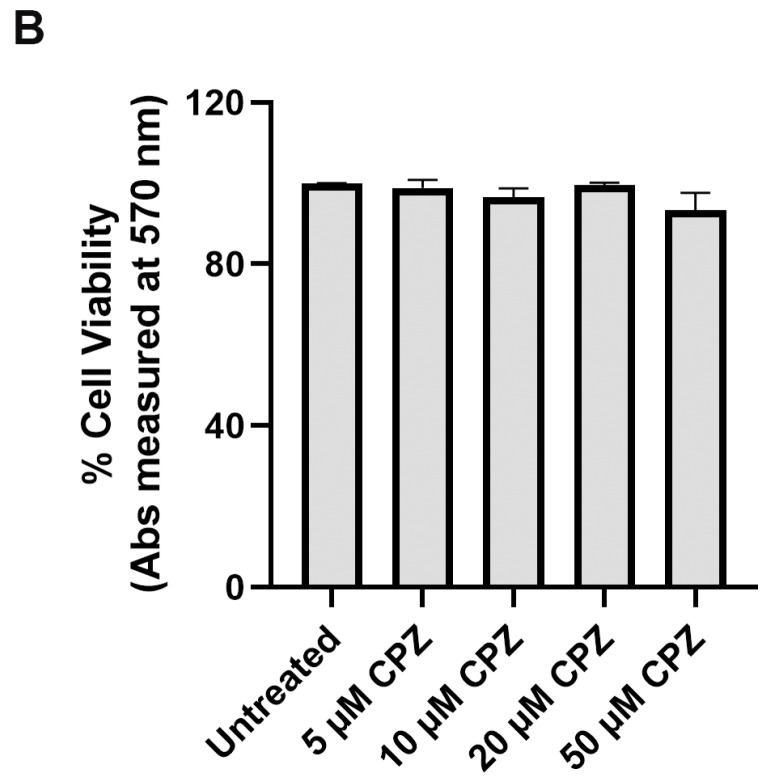
(**A**) Fold difference in uptake of original spike-pseudotyped lentiviruses in the presence of 5–50 μM chlorpromazine (CPZ) at 24 h (□) and 48 h (■) compared to untreated controls infected with original spike protein pseudotyped particles at MOI 10 alone. (**B**) CPZ is not toxic to ARPE-19: % cell viability in MTT assay at concentrations of CPZ. ** *p* < 0.001, *** *p* < 0.0005.

We utilized chlorpromazine (CPZ), a cationic amphipathic drug that is used in the micromolar range (50–100 µM) to inhibit the CME of various plasma membrane proteins. However, ARPE-19 cells are sensitive to this drug, so we could use only up to a 50 µM concentration. We saw an inhibitory effect on infection at this concentration at the 24 h timepoint but not at the 48 h timepoint (Figure 8A,B). We could not use phenylarsine oxide to inhibit CME due to cell death at the effective concentrations of this inhibitor. (higher than 5 µM; Supplemental Figure S4). In addition to chlorpromazine, which inhibits CME and dynamin GTPase activities, we investigated flunarizine, a T-type Ca^2+^ channel blocker, which inhibits lipid-stimulated dynamin I (IC_50_ = 14.1 μM) and dynamin II (IC_50_ = 0.65 μM) GTPase activities but not CME [51]. We observed only modest inhibition of spike pseudovirions infection (Supplemental Figure S4). On the other hand, dynasore, a non-competitive inhibitor of dynamin GTPase activity that has wider effects on cellular cholesterol, lipid rafts, and actin [52], demonstrated a larger inhibitory effect on infection in our system (Supplemental Figure S5).

**Figure 9:**
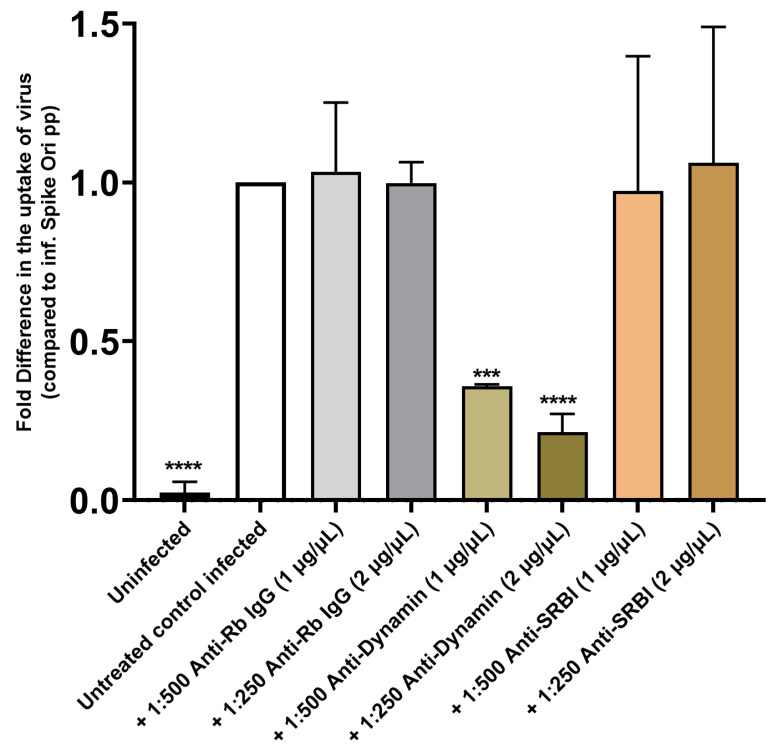
Antibody-mediated inhibition of dynamin I/II but not SRB1 blocks S protein pseudovirion infection of ARPE-19 cells. Fold difference in uptake of original spike-pseudotyped lentiviruses at MOI 10 in the presence of anti-dynamin I/II, anti-SRB1, and anti-rabbit IgG fraction (negative control) antibodies at 1:500 dilution (1 µg/mL) and 1:250 dilution (2 µg/mL) at 48 h compared to untreated controls infected with original spike protein pseudotyped particles alone. *** *p* < 0.0005, **** *p* < 0.0001.

In contrast, using anti-dynamin antibodies, we found that internalization of SARS-CoV-2 pseudovirions was mostly dynamin-dependent in our system (Figure 9). However, inhibitors of dynamin GTPase activity (flunarizine and dynasore) demonstrated less effect on infection than anti-Dynamin I/II antibodies (Supplemental Figures S4 and S5). It seems that the pleiotropic effects of dynamin and CME inhibitors might complicate the analysis of the endocytosis of spike-pseudotyped lentiviral particles.

**Figure 10:**
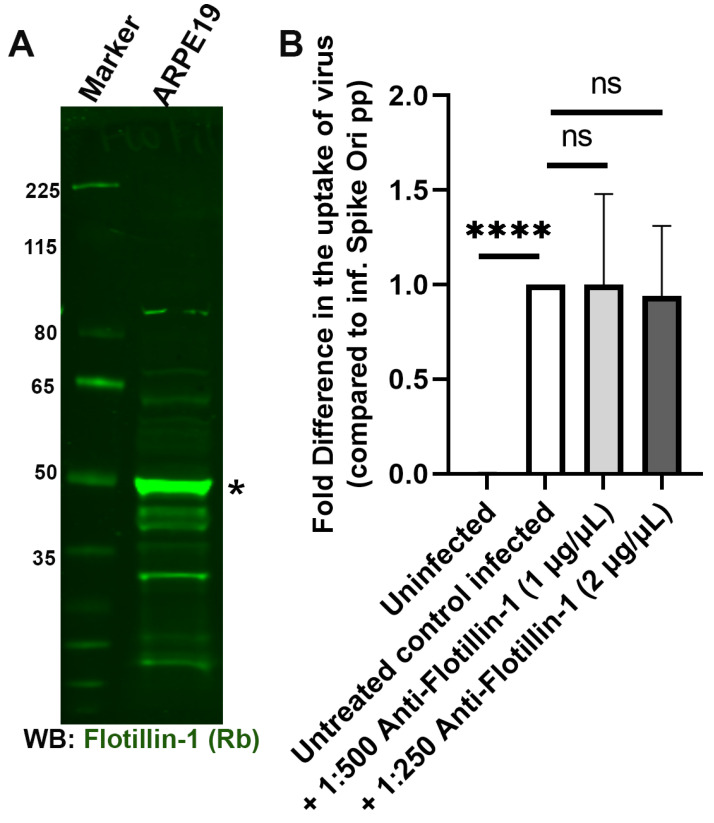
Antibody-mediated blocking of flotillin-1 has no effect in blocking S protein pseudovirion infection of ARPE-19 cells. (**A**) Flotillin-1 is expressed in ARPE-19 cells; immunoblot visualized with anti-flotillin-1 antibody (*; green). (**B**) Fold difference in uptake of original spike-pseudotyped lentiviruses at MOI 10 in the presence of anti-flotillin antibody at 1:500 dilution (1 µg/mL) and 1:250 dilution (2 µg/mL) at 24 h compared to untreated controls infected with original spike protein pseudotyped particles alone. **** *p* < 0.0001, ns—*p* > 0.05.

Another possible avenue for viruses to enter cells is via the involvement of flotillin-dependent lipid rafts [53,54]. Lipid rafts have been implicated in SARS-CoV-2 endocytosis [25,55,56]. However, we determined that anti-flotillin I antibodies do not block infection of ARPE-19 by pseudoviral particles, though flotillin is well expressed in ARPE-19 cells (Figure 10).

### 2.4. Caveolae-Mediated LDL Transcytosis Is Highjacked by Spike Pseudotyped Lentivral Particles to Infect ARPE19 Cells in Our SARS-CoV-2 Infection Model

**Figure 11:**
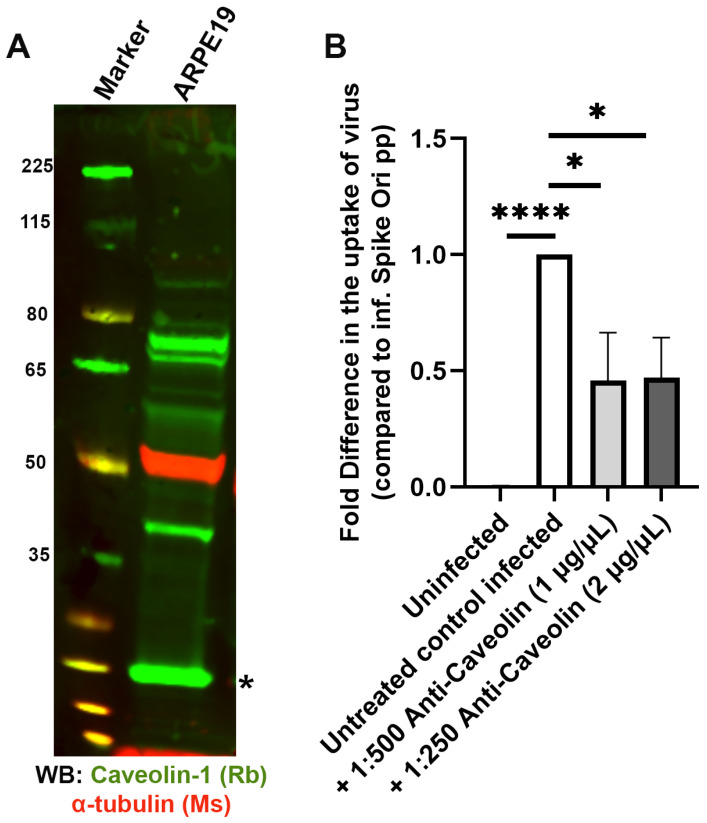
Antibody-mediated inhibition of caveolin-1 blocks S protein pseudovirion infection of ARPE-19 cells. (**A**) Caveolin-1 is expressed in ARPE-19 cells; immunoblot visualized with anti-caveolin-1 antibody (*; green). (**B**) Fold difference in uptake of original spike-pseudotyped lentiviruses at MOI 10 in the presence of Anti-caveolin-1 antibody (PA1-064) at 1:500 dilution (1 µg/mL) and 1:250 dilution (2 µg/mL) at 24 h compared to untreated controls infected with original spike protein pseudotyped particles alone. * *p*  <  0.01, **** *p* < 0.0001.

Next, we probed caveolae-dependent endocytosis with an anti-caveolin-1 antibody and established that anti-caveolin-1 significantly blocks spike pseudotyped lentiviral infection of ARPE-19 (Figure 11).

**Figure 12:**
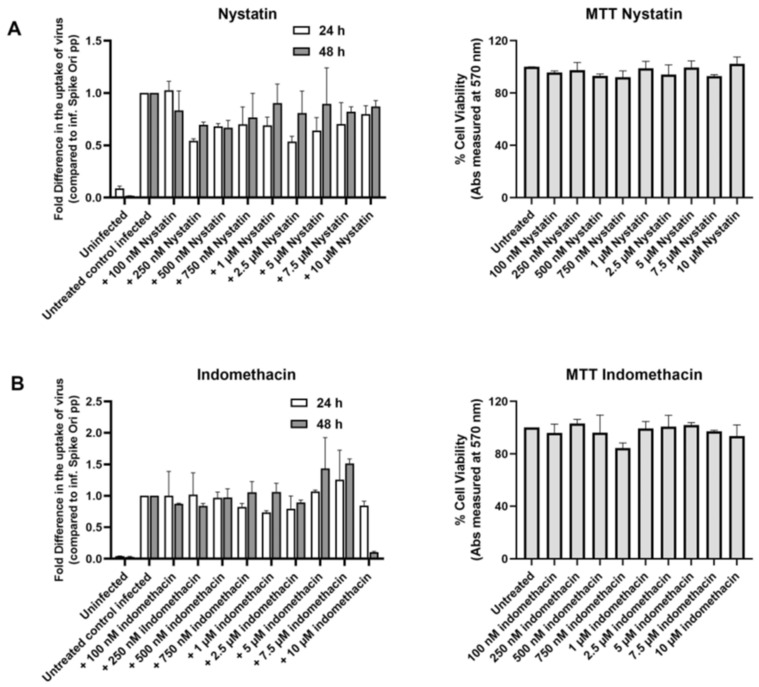
Classic inhibitors of caveolae-dependent endocytosis do not affect S protein pseudovirion infection of ARPE-19 cells. (**A**) Left panel: Fold difference in uptake of original spike-pseudotyped lentiviruses in the presence of 100 nM–10 μM nystatin at 24 h (□) and 48 h (■) compared to untreated controls infected with original spike protein pseudotyped particles only. Right panel: % Cell viability in MTT assay at various concentrations of nystatin. (**B**) Left panel: Fold difference in uptake of original spike-pseudotyped lentiviruses at MOI 10 in the presence of 100 nM to 10 μM indomethacin at 24 h (□) and 48 h (■) compared to untreated controls infected with original spike protein pseudotyped particles alone. Right panel: % Cell viability in MTT assay at various concentrations of indomethacin.

However, when we tested the classic inhibitors of caveolae-dependent endocytosis, we found that nystatin did not affect infection (Figure 12A), while indomethacin inhibited only at the highest 10 µM concentration at the 48 h time point (Figure 12B).

**Figure 13:**
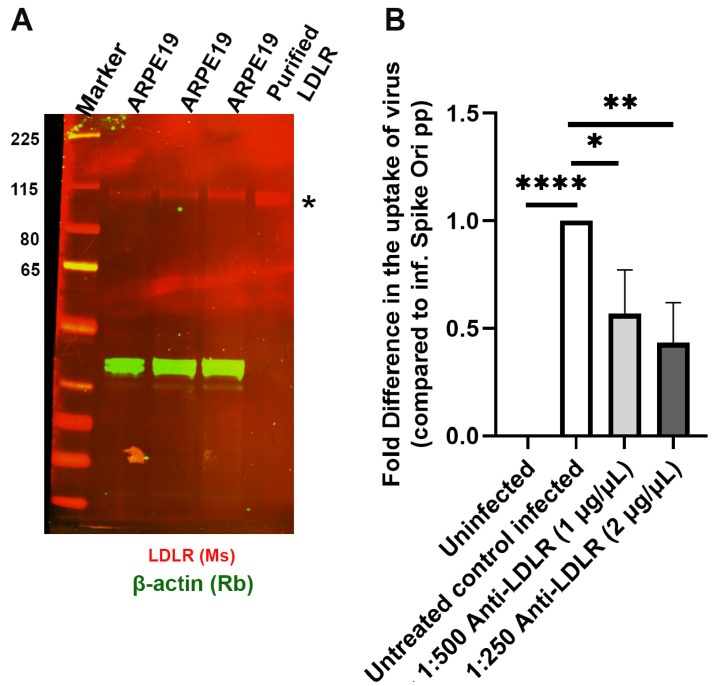
Antibody-mediated inhibition of LDLR blocks S protein pseudovirion infection of ARPE-19 cells. (**A**). LDLR is expressed in ARPE-19 cells; immunoblot visualized with anti-LDLR antibody (*; red). (**B**). Fold difference in uptake of original spike-pseudotyped lentiviruses at MOI 10 in the presence of anti-LDLR antibody at 1:500 dilution (1 µg/mL) and 1:250 dilution (2 µg/mL) at 24 h compared to untreated controls infected with original spike protein pseudotyped particles alone. * *p* < 0.01, ** *p* < 0.001, **** *p* < 0.0001.

Xiang-Li Bai and co-authors have described caveolae-mediated LDL transcytosis in endothelial cells [57], and it has been computationally predicted that the LDLR receptor could bind the SARS-CoV-2 spike protein [16]. We tested the LDLR hypothesis with anti-LDLR antibodies and found that two types of anti-LDLR antibodies strongly inhibited SARS-CoV-2 pseudovirion entry into ARPE-19 cells to a similar degree as anti-caveolin-1 and anti-Dynamin I/II antibodies (Figure 13).

**Figure 14:**
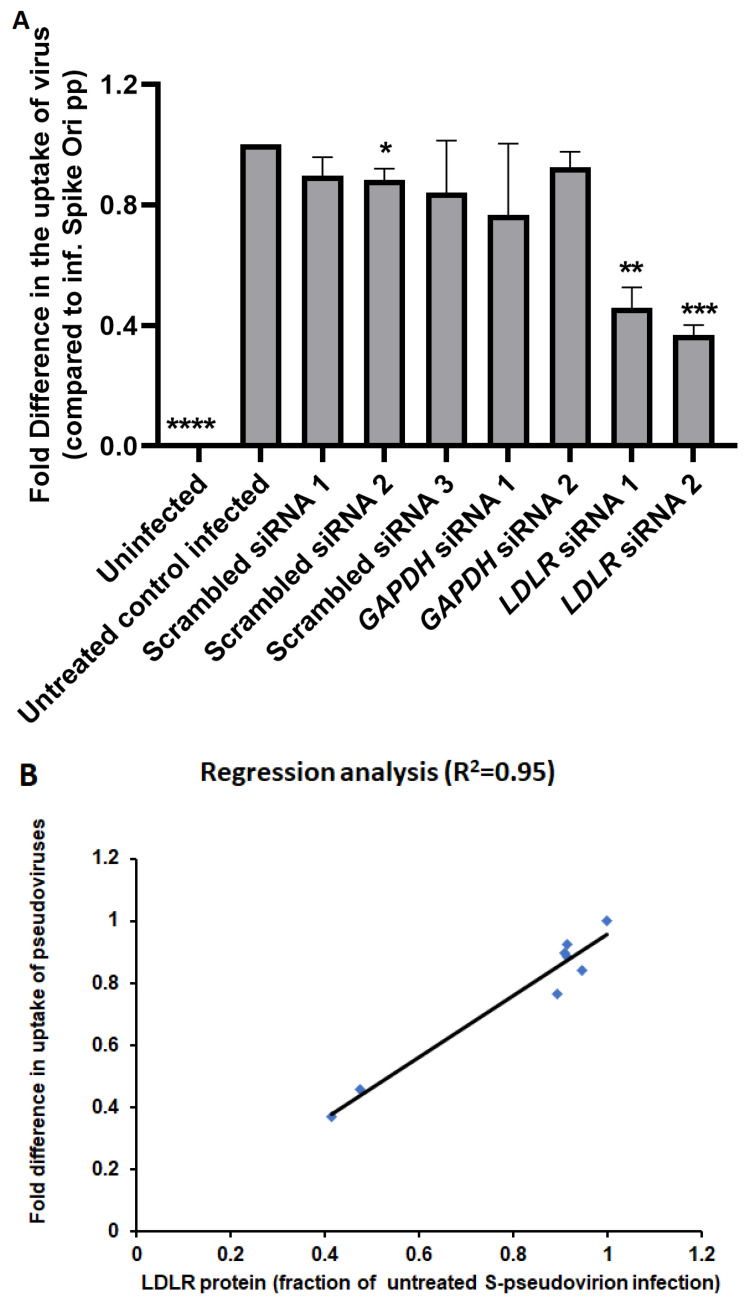
(**A**) Knockdown of LDLR expression by siRNA impairs S protein pseudovirion infection of ARPE-19 cells. ARPE-19 cells were transfected with siRNAs for LDLR, GAPDH, scrambled (1 and 2), and LDLR-scrambled and infected with SARS-CoV-2 spike pseudotyped virus at MOI 10 for 24 h. Microscopy images were taken at 72 h post-infection. The graph shows the fold difference in the uptake of original spike-pseudotyped lentiviruses in the presence of various siRNAs (25 pmol; Supplementary Table S1), compared to untreated controls infected with original spike protein pseudotyped particles alone. * *p* < 0.01, ** *p* < 0.001, *** *p* < 0.0005, **** *p* < 0.0001 in unpaired with unequal variances student’s *t*-test. (**B**) Regression analysis for LDLR protein amount in ARPE-19 samples and uptake of S-protein pseudotyped lentiviruses by ARPE-19 cells.

On the other hand, anti-SRB1 antibodies did not affect the level of infection (Figure 9). The SRB1 receptor is involved in reverse cholesterol transport and has been proposed to facilitate ACE2-dependent entry of SARS-CoV-2 [15]. Therefore, we conclude that spike pseudotyped infection is a dynamin-dependent process that is mostly mediated by the LDLR receptor in ARPE-19 cells. These findings were further confirmed by gene silencing using LDLR-specific siRNAs. We observed a significant decrease in spike pseudotyped lentiviral infection after treatment with LDLR siRNAs compared to blank transfection or scrambled siRNAs (Figure 14A, Supplementary Table S1). Furthermore, we found that the amount of LDLR protein expressed in cells is linearly correlated (R^2^ = 0.95) with the uptake of spike-pseudotyped lentiviral particles (Figure 14B and Figure S6).

### 2.5. LDL Receptor Overexpression in HeLa (hLDLR-HeLa) Greatly Increased Spike Pseudotyped Lentivral Particles Uptake in Our Model of SARS-CoV-2 Infection

**Figure 15:**
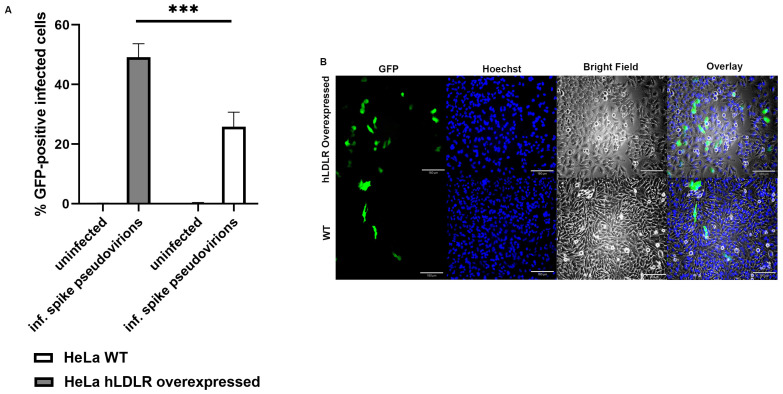
(**A**). Overexpression of LDLR in hLDLR-HeLa cells significantly increases S protein pseudovirion infection of ARPE-19 cells (*p* < 0.0005). The graph shows the fold difference in the uptake of original spike-pseudotyped lentiviruses in HeLa cells compared to stably expressing hLDLR-HeLa cells. *** *p* <  0.0005. (**B**) GFP fluorescence (green) analysis of infected hLDLR-HeLa (top panels) and HeLa cells (bottom panels) with Expi293-produced original SARS-CoV-2 spike pseudovirions, Hoechst nuclear staining (blue), bright field image, and overlay of all three images (from left to right). 4× magnification and scale bar: 180 µm.

Using a previously developed HeLa cell line stably transfected with hLDLR [58], we found that overexpression of hLDLR increases spike-pseudotyped lentiviral infection by several folds (Figure 15A,B).

### 2.6. Modes of Evolution of ACE2 and LDLR

Host-virus arms races are proposed to drive evolution in cellular viral receptors [59]. To evaluate adaptive evolution in SARS-CoV-2 viral receptors in response to viral infection, we quantified positive selection for ACE2 and LDLR receptors.

Positive (diversifying) selection is well known for many immunity- and defense-related genes, which are involved in dynamic interactions with pathogens [60]. As a category, these genes have experienced by far the most positive evolutionary selection in humans and other organisms [60]. To analyze LDLR and ACE2 (ACE2 is well known to interact with SARS and SARS-CoV-2), we performed analyses of multiple alignments of these genes in primates.

The FEL approach [61] detected no positions in the LDLR and ACE2 sequence alignment that are likely to experience episodic positive selection (positions with *p* < 0.05; Supplementary Tables S2A and S3A). However, many positions have *p* > 0.95, which suggests frequent purifying selection suggesting strong conservation of LDLR and ACE2 in the evolution of primates.

The MEME program [62] detected 10 positions in the LDLR sequence alignment that are likely to experience episodic positive selection (Supplementary Tables S2B and S3B). Similar results were obtained for ACE2 (18 sites that are likely to experience positive selection: Supplementary Tables S2 and S3). The difference between the numbers of positively selected sites in LDLR and ACE2 is not statistically significant (*p* = 0.092, Fisher exact test). The FEL [61] approach is known to produce more conservative results compared to MEME [61,62], so some differences in results between FEL and MEME are expected.

## 3. Discussion

The SARS-CoV-2 pandemic has presented a unique challenge as a rapidly moving and changing viral target has infected the global population. So far, according to data collected by Johns Hopkins University, it has taken the lives of over 5 million people worldwide. Since the beginning of the pandemic, a central question has been how the virus is internalized by human cells. It was determined that ACE2 is the canonical and major pathway for the SARS-CoV-2 virus to infect cells. However, it has been found that certain cell types have very low levels of ACE2 expression, yet SARS-CoV-2 can still infect them. Clearly, the SARS-CoV-2 virus uses several other cell surface receptors besides ACE2 and TMPRSS2, the most well-known, to enter different cell types. We are interested in how viruses gain entry into ocular cells and chose the cultured human retinal pigment epithelium cell model ARPE-19 to investigate this process. Here, we present evidence that caveolae-mediated endocytosis via LDLR receptor is the major pathway for SARS-CoV-2 virus internalization in ARPE-19 cells. We observed that entry was mostly a dynamin-dependent process with clear caveolin-1- and LDLR-mediated uptake while other dynamin-dependent processes like CME could not be ruled out, especially because CME utilizes LDLR too [63]. It is difficult to pursue CME involvement in detail because of the toxicity of ARPE-19 cells and the pleiotropic effects of endocytosis inhibitors.

Recently, direct binding of the SARS-CoV-2 spike protein to cholesterol was documented [15]. Here we demonstrate that SARS-CoV-2 pseudovirions depend on membrane cholesterol for entry into ARPE-19 cells. We propose that the SARS-CoV-2 spike protein binds to the LDL receptor and that the entire virus is endocytosed with the assistance of caveolae proteins and shuttled into early/recycling endosomes. We suspect that the virus hijacks cholesterol-recycling (LDL transcytosis) between endosomes and ER in cells and that it is delivered to the ER without processing in lysosomes. In this regard, we found that Bafilomycin A1 blocks ARPE-19 virus uptake. While Bafilomycin A1 is a well-known agent for lysosomal alkalinization, it can also alkalinize endosomal compartments and impair their activity [64]. Caveolin-mediated endocytosis of viruses by hosts requires the fusion of pseudovirion with endosomal membrane for cytoplasmic delivery of the viral genome (https://viralzone.expasy.org/976, accessed on 16 July 2023). We were able to corroborate our findings with the demonstration of an increase in the uptake of spike-pseudotyped pseudovirions by hLDLR-HeLa cells compared to unmodified HeLa cells.

Analysis of ACE2 and LDLR alignments detected no positively selected sites (the FEL method) or small numbers of positively selected sites (the MEME approach) (https://stevenweaver.github.io/hyphy-site/methods/selection-methods/, accessed on 27 June 2021). These results suggest that the major modes of evolution are purifying selection or neutral evolution (e.g., synonymous mutations), most likely due to the evolutionary conservation of the main functions of ACE2 and LDLR (interactions with host metabolites). The dominant mode of selection is purifying selection, as reflected by the effective absence of frequent episodic positive selection. Thus, how evolutionary conflicts are resolved between the main functions of ACE2 and LDLR and their episodic interactions with viruses are similar for both receptors. This conclusion is consistent with the hypothesis that both receptors are likely to be involved in the internalization of various viruses similarly.

Overall, our results suggest that caveolae-mediated transcytosis of viruses associated with the LDLR receptor is likely to be an alternative pathway for SARS-CoV-2 virus internalization in cells with low ACE2 expression, including ocular cells such as ARPE-19. Recently, it was demonstrated that white adipose tissue (WAT) surface expression of LDL receptor (LDLR) and/or CD36 is associated with metabolic dysfunction and insulin resistance, both WAT-directed and systemic [65]. We speculate that LDLR receptor involvement in metabolic dysfunction could translate to a higher risk of developing serious COVID disease in obese and diabetic patients and warrant further investigation.

## 4. Materials and Methods

### 4.1. Antibodies, Inhibitors, and Reagents

Bafilomycin-A1 (Cat. No. 54645) and Lovastatin, HMG-CoA reductase inhibitor (Cat. No. BML-G226-0010), were purchased from Enzo Life Sciences (Farmingdale, NY, USA). α-cyclodextrin (αCD, Cat. No. C4680), methyl β-cyclodextrin (MβCD, Cat. No. C4555), β-cyclodextrin (βCD, Cat. No. C4805), (2-hydroxypropyl)-β-cyclodextrin (2HPβCD, Cat. No. 332593), phenylarsine oxide (PAO), Cat. No. P3075), chlorpromazine hydrochloride (CPZ) (Cat. No. C8138), Indomethacin (Cat. No. I7378), Nystatin (Cat. No. N6261), GW3965 hydrochloride (Cat. No. G6295) from Millipore Sigma (Burlington, MA, USA).

### 4.2. Cells and Culture

Expi293F™ suspension cells were grown according to the manufacturer’s instructions (Thermo Fisher Scientific, Waltham, MA, USA), and production of SARS-CoV-2 spike protein pseudoviruses was carried out using Expi293F™ Expression Medium. To examine the infection of the SARS-CoV-2 spike protein pseudovirions, we used earlier passages of the ARPE-19 cell line [66], following our previous experiments [67]. ARPE-19 cells were maintained in DMEM media with 5% FBS (GeminiBio, West Sacramento, CA, USA), 1% sodium pyruvate, and 1% penicillin-streptomycin supplementation and cultured at 37 °C in a 5% CO_2_ incubator. For siRNA experiments, cells were maintained on DMEM media with 1% FBS, 1% sodium pyruvate, and 1% penicillin-streptomycin supplementation.

HeLa cells and hLDLR-HeLa cells were seeded at 5 × 10^4^ density (into 12 well plates) and maintained on DMEM + Glutamax with 1% penicillin-streptomycin (Thermo Fisher Scientific, Waltham, MA, USA) and 10% FBS (GeminiBio, West Sacramento, CA, USA).

### 4.3. Immunoblot Analysis

Immunoblotting was performed as described previously [25]. In brief, ARPE-19 cells infected with S-protein pseudotyped lentiviral particles were harvested 48 h post-transfection by centrifuging at 1500× *g* for 10 min at room temperature. The cell pellets were lysed using RIPA buffer (20 mM Tris-HCl pH 7.5, 150 mM NaCl, 1 mM EDTA, 0.1% SDS, 1% NP-40, and 1× protease inhibitor cocktail), sonicated, and centrifuged at 16,000× *g* for 10 min to clear the nuclear debris, and the supernatant (total cell lysate) was collected in a clear tube. Protein estimation was performed using Pierce™ Coomassie Plus (Bradford) Assay Kit (Thermo Fisher Scientific). Samples were prepared in 4× LDS buffer and heated at 95 °C for 10 min. Then, 30 μg of total protein was loaded on Invitrogen 4–12% Bolt Plus Bis-Tris gels (Thermo Fisher Scientific) and subjected to immunoblotting for calreticulin (1:2000 dilution, Goat mAb; Abcam, Cambridge, MA, USA) and various cell receptors (Table 1). Secondary antibodies (LI-COR Biosciences, Lincoln, NE, USA, 1:15,000 dilution) in Intercept™ Blocking buffer (LI-COR Biosciences, Lincoln, NE, USA) were used. Membranes were scanned on an Odyssey Infrared Imager (LI-COR Biosciences, Lincoln, NE, USA) and image data were processed using Image Studio™ Lite V3.1 (LI-COR Biosciences, Lincoln, NE, USA).

### 4.4. Production of SARS-CoV-2 Spike Protein Pseudovirions

SARS-CoV-2 Spike protein pseudovirions were produced by co-transfection of Expi293F™ cells (Cat. # A14527, Thermo Fisher Scientific, Waltham, MA USA) with packaging plasmid psPAX2, pLenti-GFP transfer plasmid, and SARS-CoV-2 S protein (original sequence) in a ratio of 3:2:2, respectively. A total of 30 µg DNA was used to transfect 30 mL of cell culture using the 293fectin™ transfection reagent (Thermo Fisher Scientific, Waltham, MA USA). The supernatant was harvested 48 h post transfection, centrifuged at 800× *g* for 5 min, and passed through 0.45 µm PES membrane filters (Genesee Scientific, Morrisville, NC, USA). Pseudotyped virus stocks were aliquoted and stored in cryovials at −80 °C.

#### 4.4.1. Measurement of Physical and Infectious Viral Titer

The physical viral titer was measured as described previously [25]. Briefly, RNA was extracted using Maxwell RCS Viral TNA (Promega AS1330, Madison, WI, USA), followed by turbo DNase treatment (Invitrogen/Thermo Fisher Scientific AM1907, Waltham, MA, USA). cDNA was synthesized (High-Capacity cDNA Reverse Transcription Kit, Thermo Fisher Scientific, Waltham, MA USA) and used for TaqMan qPCR with primers for viral LTR and WPRE. Standard curves were obtained using the pLenti plasmid.

To measure the infectivity of viral titer, ARPE-19 cells were seeded at a cell density of 2–5 × 10^4^ in 24-well plates and infected with different volumes of pseudotyped virus. GFP fluorescence in the infected cells was visualized using a Revolve microscope (Discover Echo, San Diego, CA, USA) with a 10× objective. At 48 and 72 h after transduction, the percentages of GFP-positive cells were measured using a Cytation instrument (Model CYT7UW, BioTek, Winooski, VT, USA). The total cell count per well was identified using a high-contrast mask on brightfield images. GFP-positive cells were identified using a mask on fluorescent images using Gen5 Image Prime Software Version 3.10 (BioTek, Winooski, VT, USA).

#### 4.4.2. Pseudovirus Infection Assay

For this process, 5 × 10^4^ ARPE-19 cells in 1 mL DMEM per well were seeded into 24-well plates. The cells were cultured in a 37 °C, 5% CO_2_ incubator for 24 h. The medium was aspirated, and fresh medium containing the 100 µL pseudoviruses (5 × 10^5^ viral particles; MOI 10) in the absence and presence of antibodies was added and incubated in a 37 °C 5% CO_2_ incubator for 24 h. After 24 h, the culture medium containing the virus-antibody mixture was removed and replaced by 1000 μL of fresh DMEM, and incubated continuously at 37 °C for 48 h.

In the case of the use of chemical inhibitors, ARPE-19 cells were pre-treated with different concentrations of inhibitors for 4–6 h, and then the medium was aspirated and fresh medium containing the 100 µL pseudoviruses was added and incubated at 37 °C in a humidified atmosphere of 5% CO_2_ for 16 h. Subsequently, the unbound pseudoviruses were removed by aspirating the media and replaced with a fresh DMEM medium. For GFP fluorescence intensity measurements, the media was changed to no phenol red media. At different time points, the GFP fluorescence intensity was measured using the Cytation instrument.

HeLa and hLDLR-HeLa cells in 2 mL of media were seeded at 5 × 10^4^ density into 12 well plates. The cells were cultured in a 37 °C/5% CO_2_ incubator for 24 h and then infected with 5 × 10^5^ viral particles (MOI 10) for 48 h. After that, the GFP fluorescence intensity was measured using the Cytation instrument (Model CYT7UW, BioTek, Winooski, VT, USA).

### 4.5. Cell Viability Assay

Cell viability was evaluated using a CyQUANT™ MTT cell proliferation assay kit 8 (Cat. # V13154; Invitrogen, Waltham, MA, USA) following the manufacturer’s instructions. Briefly, ARPE-19 cells were seeded into 24-well plates at a density of 5 × 10^4^ cells per well. The next day, the cells were treated with different concentrations of chemical inhibitors for 4–6 h. Then, the medium was replaced with 300 µL DMEM (without phenol red supplemented with 5% FBS and 1% sodium pyruvate) containing 6 µL of the 12 mM MTT stock solution per well. A negative control was included in a well without cells, containing 6 µL of the MTT stock solution per 300 µL of the medium alone. The microplates were incubated overnight at 37 °C in a humified chamber, and the next day, 100 µL of SDS-HCl solution was added to each well. Microplate was further incubated for 4 h at 37 °C and the samples were mixed by pipetting up and down before reading the plate at an absorbance of 570 nm using a SpectraMax iD5 Multi-mode Microplate Reader (Molecular Devices, San Jose, CA, USA). The percentage of viable cells was calculated using the formula: [(OD_Treated_ − OD_Blank_)/(OD_Control_ − OD_Blank_)] × 100%. Each sample was assayed with three replicates per measurement.

### 4.6. siRNA Treatment and Pseudovirion Infection of ARPE-19 Cells

Pre-designed siRNAs against LDLR (Ambion™ Silencer™ si-LDLR-1: siRNA ID# 110672; and si-LDLR-2: siRNA ID# 106132) were ordered from ThermoFisher Scientific (Waltham, MA USA). The scrambled control siRNA #1, siRNA #2, and siRNA #3 were designed previously [68] (Supplementary Table S3). Briefly, ARPE-19 cells (cell density of 3 × 10^5^ cells/well) were seeded in six-well plates, and siRNA (25 pmol/well) was mixed with Lipofectamine™ RNAiMAX (Waltham, MA, USA, 7.5 µL/well) transfection reagent. A total of 250 µL of opti-MEM (ThermoFisher Scientific, Waltham, MA USA) was added to the cells, and the cells were incubated for 72 h.

To analyze the silencing of LDLR protein levels, cells were harvested after 72 h of siRNA treatment, washed with ice-cold 1× PBS buffer, and resuspended in 200 µL of RIPA lysis buffer with inhibitors. The cell lysate was incubated for 15 min on ice, sonicated three times (2 s ON/1 min OFF), and centrifuged at 13,000× *g* for 5 min at 4 °C. The supernatant was collected, and protein concentration was determined by Bradford protein assay using Pierce Coomassie Plus Assay Reagent (ThermoFisher Scientific, Waltham, MA USA). Samples were prepared in 4× SDS loading dye, heated at 95 °C, centrifuged, and subjected to SDS-PAGE followed by western blotting analysis with anti-LDLR antibody. The experiments were repeated at least three times.

For pseudovirus infection experiments, we added 200 µL of SARS-CoV-2 spike pseudoviruses after 48 h of siRNA treatment and incubated for 24 h. The next day, the medium containing siRNAs and pseudoviruses was aspirated, replaced with fresh medium without phenol red, and incubated in a 37 °C incubator containing 5% CO_2_ for another 72 h. After 72 h, the GFP fluorescence intensity was measured using the Cytation instrument to determine the % of GFP-expressing infected cells.

### 4.7. Multiple Alignments Analyses

We performed analyses of multiple alignments of primate, dog, and mouse LDLR and ACE2 sequences downloaded from the University of California at Santa Cruz Table Browser (hgdownload.cse.ucsc.edu/goldenpath/hg38/multiz30way/alignments/, accessed on 3 February 2022). FEL (Fixed Effects Likelihood) and MEME (Mixed Effects Model of Evolution) approaches (implemented in the HyPhy package; https://stevenweaver.github.io/hyphy-site/methods/selection-methods/, accessed on 27 June 2021) were used for the detection of sequence positions that are likely to experience positive selection. The FEL [61] approach is known to produce more conservative results compared to the MEME approach [61,62].

### 4.8. Statistical Analysis of Data

The numbers of positively selected sites vs. the numbers of remaining sites were compared using the 2 × 2 Fisher exact test, with a significant difference at *p* < 0.05.

Results were calculated in Excel 365 as mean ± S.D. from three independent experiments. * *p* < 0.01, ** *p* < 0.001, *** *p* < 0.0005, **** *p* < 0.0001, and ns—*p* > 0.05 unpaired with unequal variances student’s *t*-test were performed in GraphPad 9.3.1.

## Figures and Tables

**Table 1:** Antibodies used in experiments.

Antibody	Catalog No.	Company Name
Normal Mouse IgG (1 mg/mL)	12-371	Millipore Sigma (Burlington, MA, USA)
Normal Rabbit IgG (1 mg/mL)	12-370	Millipore Sigma (Burlington, MA, USA)
Mouse anti-EGFR monoclonal clone LA22	05-104	Millipore Sigma (Burlington, MA, USA)
ACE-2 mouse monoclonal IgG_2A_ clone 171606	MAB933	R&D Systems (Minneapolis, MN, USA)
ACE-2 mouse monoclonal IgG_2A_ clone 535919	MAB9332-100	R&D Systems (Minneapolis, MN, USA)
ACE-2 goat polyclonal IgG (0.2 mg/mL)	AF933	R&D Systems (Minneapolis, MN, USA)
Mouse anti-human monoclonal Neuropilin-1 (0.34 mg/mL)	MAB3870	R&D Systems (Minneapolis, MN, USA)
Mouse monoclonal Ultra-Leaf™ Purified anti-human CD147 (2 mg/mL)	306221	Biolegend^®^ (San Diego, CA, USA)
Axl rabbit polyclonal IgG (0.34 mg/mL)	PA5-34658	Thermo Fisher Scientific (Waltham, MA, USA)
Caveolin-1 rabbit polyclonal IgG (1 mg/mL)	PA1-064	Thermo Fisher Scientific (Waltham, MA, USA)
Vimentin (V9) mouse monoclonal (0.5 mg/mL)	sc-6260	Santa Cruz Biotechnology, Inc. (Dallas, TX, USA)
Vimentin chicken polyclonal IgY	NB-300-223	Novus Biologicals (Centennial, CO, USA)
Dynamin I/II rabbit polyclonal IgG	2342	Cell Signaling Technology^®^ (Danvers, MA, USA)
Flotilin-1 rabbit polyclonal IgG (0.34 mg/mL)	3253	Cell Signaling Technology^®^ (Danvers, MA, USA)
Clathrin HC (C-20) goat polyclonal IgG (200 µg/mL)	sc-6579	Santa Cruz Biotechnology, Inc. (Dallas, TX, USA)
LDL Receptor Mouse monoclonal [1B10H10] antibody (1 mg/mL)	ab204941	Abcam (Waltham, MA, USA)
LDL Receptor Mouse monoclonal clone C7	MABS26	Millipore Sigma (Burlington, MA, USA)
SR-B1 rabbit polyclonal IgG	NB400-113	Novus Biologicals (Centennial, CO, USA)
Secondary IRDye 800CW Goat Anti-Rabbit	925-32211	LI-COR Biosciences, Lincoln, NE, USA
Secondary IRDye 680RD Goat Anti-Mouse	926-68070	LI-COR Biosciences, Lincoln, NE, USA
Secondary IRDye 680RD Donkey Anti-Goat	925-68074	LI-COR Biosciences, Lincoln, NE, USA

## Data Availability

The data presented in this study are available in the article and Supplementary Material.

## Supplementary Materials

The supporting information can be downloaded at: https://www.mdpi.com/article/10.3390/ijms241411860/s1.

## Abbreviations

ACE2 angiotensin-converting enzyme 2; α-CD, α-cyclodextrin; AT1, Angiotensin II receptor type 1; AVPR1B, Vasopressin V1b receptor; β-CD, β-cyclodextrin; CD147, Basigin; CME, clathrin-mediated endocytosis; CPZ, chlorpromazine; FBS, fetal bovine serum; h, hours; 25-HC, 25-hydroxycholesterol; hLDLR-HeLa, human LDL receptor overexpression in HeLa; 2-HPβ-CD, 2-Hydroxypropyl-β-cyclodextrin; HRV, human rotavirus; Inf. Spike ori pp, infection with original Wuhan strain SARS-CoV-2 spike protein pseudotyped particles; LDLR, Low-density lipoprotein receptor; mAbs, monoclonal antibodies; Mβ-CD, methyl-β-cyclodextrin; MOI, multiplicity of infection; NRP-1, Neuropilin-1; PAO, phenylarsine oxide; siRNA, small interfering RNA.

## References

[1] Walls A.C., Park Y.J., Tortorici M.A., Wall A., McGuire A.T., Veesler D. (2020). Structure, Function, and Antigenicity of the SARS-CoV-2 Spike Glycoprotein. Cell.

[2] Zhou P., Yang X.L., Wang X.G., Hu B., Zhang L., Zhang W., Si H.R., Zhu Y., Li B., Huang C.L. (2020). A pneumonia outbreak associated with a new coronavirus of probable bat origin. Nature.

[3] Wang Q., Zhang Y., Wu L., Niu S., Song C., Zhang Z., Lu G., Qiao C., Hu Y., Yuen K.Y. (2020). Structural and Functional Basis of SARS-CoV-2 Entry by Using Human ACE2. Cell.

[4] Yan R., Zhang Y., Li Y., Xia L., Guo Y., Zhou Q. (2020). Structural basis for the recognition of SARS-CoV-2 by full-length human ACE2. Science.

[5] Hoffmann M., Kleine-Weber H., Schroeder S., Kruger N., Herrler T., Erichsen S., Schiergens T.S., Herrler G., Wu N.H., Nitsche A. (2020). SARS-CoV-2 Cell Entry Depends on ACE2 and TMPRSS2 and Is Blocked by a Clinically Proven Protease Inhibitor. Cell.

[6] Eriksen A.Z., Moller R., Makovoz B., Uhl S.A., tenOever B.R., Blenkinsop T.A. (2021). SARS-CoV-2 infects human adult donor eyes and hESC-derived ocular epithelium. Cell Stem Cell.

[7] Martinez-Colon G.J., Ratnasiri K., Chen H., Jiang S., Zanley E., Rustagi A., Verma R., Chen H., Andrews J.R., Mertz K.D. (2022). SARS-CoV-2 infection drives an inflammatory response in human adipose tissue through infection of adipocytes and macrophages. Sci. Transl. Med..

[8] Partridge L.J., Urwin L., Nicklin M.J.H., James D.C., Green L.R., Monk P.N. (2021). ACE2-Independent Interaction of SARS-CoV-2 Spike Protein with Human Epithelial Cells Is Inhibited by Unfractionated Heparin. Cells.

[9] Shen X.R., Geng R., Li Q., Chen Y., Li S.F., Wang Q., Min J., Yang Y., Li B., Jiang R.D. (2022). ACE2-independent infection of T lymphocytes by SARS-CoV-2. Signal Transduct. Target. Ther..

[10] Borsa M., Mazet J.M. (2020). Attacking the defence: SARS-CoV-2 can infect immune cells. Nat. Rev. Immunol..

[11] Davanzo G.G., Codo A.C., Brunetti N.S., Boldrini V., Knittel T.L., Monterio L.B., de Moraes D., Ferrari A.J.R., de Souza G.F., Muraro S.P. (2020). SARS-CoV-2 Uses CD4 to Infect T Helper Lymphocytes. medRxiv.

[12] Karthika T., Joseph J., Das V.R.A., Nair N., Charulekha P., Roji M.D., Raj V.S. (2021). SARS-CoV-2 Cellular Entry Is Independent of the ACE2 Cytoplasmic Domain Signaling. Cells.

[13] Chi X., Yan R., Zhang J., Zhang G., Zhang Y., Hao M., Zhang Z., Fan P., Dong Y., Yang Y. (2020). A neutralizing human antibody binds to the N-terminal domain of the Spike protein of SARS-CoV-2. Science.

[14] Cantuti-Castelvetri L., Ojha R., Pedro L.D., Djannatian M., Franz J., Kuivanen S., van der Meer F., Kallio K., Kaya T., Anastasina M. (2020). Neuropilin-1 facilitates SARS-CoV-2 cell entry and infectivity. Science.

[15] Wei C., Wan L., Yan Q., Wang X., Zhang J., Yang X., Zhang Y., Fan C., Li D., Deng Y. (2020). HDL-scavenger receptor B type 1 facilitates SARS-CoV-2 entry. Nat. Metab..

[16] Wang S., Qiu Z., Hou Y., Deng X., Xu W., Zheng T., Wu P., Xie S., Bian W., Zhang C. (2021). AXL is a candidate receptor for SARS-CoV-2 that promotes infection of pulmonary and bronchial epithelial cells. Cell Res..

[17] Yeung M.L., Teng J.L.L., Jia L., Zhang C., Huang C., Cai J.P., Zhou R., Chan K.H., Zhao H., Zhu L. (2021). Soluble ACE2-mediated cell entry of SARS-CoV-2 via interaction with proteins related to the renin-angiotensin system. Cell.

[18] Pia L., Rowland-Jones S. (2022). Omicron entry route. Nat. Rev. Immunol..

[19] Filippini A., D’Alessio A. (2020). Caveolae and Lipid Rafts in Endothelium: Valuable Organelles for Multiple Functions. Biomolecules.

[20] Sorice M., Misasi R., Riitano G., Manganelli V., Martellucci S., Longo A., Garofalo T., Mattei V. (2020). Targeting Lipid Rafts as a Strategy Against Coronavirus. Front. Cell Dev. Biol..

[21] Yang N., Shen H.M. (2020). Targeting the Endocytic Pathway and Autophagy Process as a Novel Therapeutic Strategy in COVID-19. Int. J. Biol. Sci..

[22] Davis G., Li K., Thankam F.G., Wilson D.R., Agrawal D.K. (2022). Ocular transmissibility of COVID-19: Possibilities and perspectives. Mol. Cell. Biochem..

[23] Petronio Petronio G., Di Marco R., Costagliola C. (2020). Do Ocular Fluids Represent a Transmission Route of SARS-CoV-2 Infection?. Front. Med..

[24] Yamasoba D., Uriu K., Plianchaisuk A., Kosugi Y., Pan L., Zahradnik J., Ito J., Sato K. (2023). Virological characteristics of the SARS-CoV-2 Omicron XBB.1.16 variant. Lancet Infect. Dis..

[25] Postnikova O.A., Uppal S., Huang W., Kane M.A., Villasmil R., Rogozin I.B., Poliakov E., Redmond T.M. (2021). The Functional Consequences of the Novel Ribosomal Pausing Site in SARS-CoV-2 Spike Glycoprotein RNA. Int. J. Mol. Sci..

[26] Wang H., Yang P., Liu K., Guo F., Zhang Y., Zhang G., Jiang C. (2008). SARS coronavirus entry into host cells through a novel clathrin- and caveolae-independent endocytic pathway. Cell Res..

[27] Amraei R., Xia C., Olejnik J., White M.R., Napoleon M.A., Lotfollahzadeh S., Hauser B.M., Schmidt A.G., Chitalia V., Muhlberger E. (2022). Extracellular vimentin is an attachment factor that facilitates SARS-CoV-2 entry into human endothelial cells. Proc. Natl. Acad. Sci. USA.

[28] Daly J.L., Simonetti B., Klein K., Chen K.E., Williamson M.K., Anton-Plagaro C., Shoemark D.K., Simon-Gracia L., Bauer M., Hollandi R. (2020). Neuropilin-1 is a host factor for SARS-CoV-2 infection. Science.

[29] Sanders D.W., Jumper C.C., Ackerman P.J., Bracha D., Donlic A., Kim H., Kenney D., Castello-Serrano I., Suzuki S., Tamura T. (2021). SARS-CoV-2 requires cholesterol for viral entry and pathological syncytia formation. Elife.

[30] Amar M.J., Kaler M., Courville A.B., Shamburek R., Sampson M., Remaley A.T. (2016). Randomized double blind clinical trial on the effect of oral alpha-cyclodextrin on serum lipids. Lipids Health Dis..

[31] Coisne C., Tilloy S., Monflier E., Wils D., Fenart L., Gosselet F. (2016). Cyclodextrins as Emerging Therapeutic Tools in the Treatment of Cholesterol-Associated Vascular and Neurodegenerative Diseases. Molecules.

[32] Vance J.E., Karten B. (2014). Niemann-Pick C disease and mobilization of lysosomal cholesterol by cyclodextrin. J. Lipid Res..

[33] Ohtani Y., Irie T., Uekama K., Fukunaga K., Pitha J. (1989). Differential effects of alpha-, beta- and gamma-cyclodextrins on human erythrocytes. Eur. J. Biochem..

[34] Yancey P.G., Rodrigueza W.V., Kilsdonk E.P., Stoudt G.W., Johnson W.J., Phillips M.C., Rothblat G.H. (1996). Cellular cholesterol efflux mediated by cyclodextrins. Demonstration Of kinetic pools and mechanism of efflux. J. Biol. Chem..

[35] Wittkowski K.M., Dadurian C., Seybold M.P., Kim H.S., Hoshino A., Lyden D. (2018). Complex polymorphisms in endocytosis genes suggest alpha-cyclodextrin as a treatment for breast cancer. PLoS ONE.

[36] El-Darzi N., Mast N., Petrov A.M., Pikuleva I.A. (2021). 2-Hydroxypropyl-beta-cyclodextrin reduces retinal cholesterol in wild-type and *Cyp27a1^−/−^ Cyp46a1^−/−^* mice with deficiency in the oxysterol production. Br. J. Pharmacol..

[37] Civra A., Francese R., Gamba P., Testa G., Cagno V., Poli G., Lembo D. (2018). 25-Hydroxycholesterol and 27-hydroxycholesterol inhibit human rotavirus infection by sequestering viral particles into late endosomes. Redox Biol..

[38] Shang C., Zhuang X., Zhang H., Li Y., Zhu Y., Lu J., Ge C., Cong J., Li T., Tian M. (2021). Inhibitors of endosomal acidification suppress SARS-CoV-2 replication and relieve viral pneumonia in hACE2 transgenic mice. Virol. J..

[39] Blanc M., Hsieh W.Y., Robertson K.A., Kropp K.A., Forster T., Shui G., Lacaze P., Watterson S., Griffiths S.J., Spann N.J. (2013). The transcription factor STAT-1 couples macrophage synthesis of 25-hydroxycholesterol to the interferon antiviral response. Immunity.

[40] Liu S.Y., Sanchez D.J., Aliyari R., Lu S., Cheng G. (2012). Systematic identification of type I and type II interferon-induced antiviral factors. Proc. Natl. Acad. Sci. USA.

[41] Moog C., Aubertin A.M., Kirn A., Luu B. (1998). Oxysterols, but not cholesterol, inhibit human immunodeficiency virus replication in vitro. Antivir. Chem. Chemother..

[42] Shibata N., Carlin A.F., Spann N.J., Saijo K., Morello C.S., McDonald J.G., Romanoski C.E., Maurya M.R., Kaikkonen M.U., Lam M.T. (2013). 25-Hydroxycholesterol activates the integrated stress response to reprogram transcription and translation in macrophages. J. Biol. Chem..

[43] Zhao J., Chen J., Li M., Chen M., Sun C. (2020). Multifaceted Functions of CH25H and 25HC to Modulate the Lipid Metabolism, Immune Responses, and Broadly Antiviral Activities. Viruses.

[44] Park K., Scott A.L. (2010). Cholesterol 25-hydroxylase production by dendritic cells and macrophages is regulated by type I interferons. J. Leukoc. Biol..

[45] Zu S., Deng Y.Q., Zhou C., Li J., Li L., Chen Q., Li X.F., Zhao H., Gold S., He J. (2020). 25-Hydroxycholesterol is a potent SARS-CoV-2 inhibitor. Cell Res..

[46] Leussink S., Aranda-Pardos I., A-Gonzalez N. (2020). Lipid metabolism as a mechanism of immunomodulation in macrophages: The role of liver X receptors. Curr. Opin. Pharmacol..

[47] Zhang L., Reue K., Fong L.G., Young S.G., Tontonoz P. (2012). Feedback regulation of cholesterol uptake by the LXR-IDOL-LDLR axis. Arterioscler. Thromb. Vasc. Biol..

[48] Sorrentino V., Nelson J.K., Maspero E., Marques A.R.A., Scheer L., Polo S., Zelcer N. (2013). The LXR-IDOL axis defines a clathrin-, caveolae-, and dynamin-independent endocytic route for LDLR internalization and lysosomal degradation. J. Lipid Res..

[49] Glebov O.O. (2020). Understanding SARS-CoV-2 endocytosis for COVID-19 drug repurposing. FEBS J..

[50] Bayati A., Kumar R., Francis V., McPherson P.S. (2021). SARS-CoV-2 infects cells after viral entry via clathrin-mediated endocytosis. J. Biol. Chem..

[51] Daniel J.A., Chau N., Abdel-Hamid M.K., Hu L., von Kleist L., Whiting A., Krishnan S., Maamary P., Joseph S.R., Simpson F. (2015). Phenothiazine-derived antipsychotic drugs inhibit dynamin and clathrin-mediated endocytosis. Traffic.

[52] Preta G., Cronin J.G., Sheldon I.M. (2015). Dynasore—not just a dynamin inhibitor. Cell Commun. Signal..

[53] McGuinn K.P., Mahoney M.G. (2014). Lipid rafts and detergent-resistant membranes in epithelial keratinocytes. Methods Mol. Biol..

[54] Rosenberger C.M., Brumell J.H., Finlay B.B. (2000). Microbial pathogenesis: Lipid rafts as pathogen portals. Curr. Biol..

[55] Palacios-Rapalo S.N., De Jesus-Gonzalez L.A., Cordero-Rivera C.D., Farfan-Morales C.N., Osuna-Ramos J.F., Martinez-Mier G., Quistian-Galvan J., Munoz-Perez A., Bernal-Dolores V., Del Angel R.M. (2021). Cholesterol-Rich Lipid Rafts as Platforms for SARS-CoV-2 Entry. Front. Immunol..

[56] Fecchi K., Anticoli S., Peruzzu D., Iessi E., Gagliardi M.C., Matarrese P., Ruggieri A. (2020). Coronavirus Interplay with Lipid Rafts and Autophagy Unveils Promising Therapeutic Targets. Front. Microbiol..

[57] Bai X.L., Yang X.Y., Li J.Y., Ye L., Jia X., Xiong Z.F., Wang Y.M., Jin S. (2017). Cavin-1 regulates caveolae-mediated LDL transcytosis: Crosstalk in an AMPK/eNOS/NF-kappaB/Sp1 loop. Oncotarget.

[58] Vishnyakova T.G., Bocharov A.V., Baranova I.N., Kurlander R., Drake S.K., Chen Z., Amar M., Sviridov D., Vaisman B., Poliakov E. (2020). SR-BI mediates neutral lipid sorting from LDL to lipid droplets and facilitates their formation. PLoS ONE.

[59] Wang W., Zhao H., Han G.Z. (2020). Host-Virus Arms Races Drive Elevated Adaptive Evolution in Viral Receptors. J. Virol..

[60] Nielsen R., Hellmann I., Hubisz M., Bustamante C., Clark A.G. (2007). Recent and ongoing selection in the human genome. Nat. Rev. Genet..

[61] Kosakovsky Pond S.L., Frost S.D. (2005). Not so different after all: A comparison of methods for detecting amino acid sites under selection. Mol. Biol. Evol..

[62] Murrell B., Wertheim J.O., Moola S., Weighill T., Scheffler K., Kosakovsky Pond S.L. (2012). Detecting individual sites subject to episodic diversifying selection. PLoS Genet..

[63] Brown M.S., Goldstein J.L. (1986). A receptor-mediated pathway for cholesterol homeostasis. Science.

[64] Johnson L.S., Dunn K.W., Pytowski B., McGraw T.E. (1993). Endosome acidification and receptor trafficking: Bafilomycin A1 slows receptor externalization by a mechanism involving the receptor’s internalization motif. Mol. Biol. Cell.

[65] Cyr Y., Bissonnette S., Lamantia V., Wassef H., Loizon E., Ngo Sock E.T., Vidal H., Mayer G., Chretien M., Faraj M. (2020). White Adipose Tissue Surface Expression of LDLR and CD36 is Associated with Risk Factors for Type 2 Diabetes in Adults with Obesity. Obesity.

[66] Dunn K.C., Aotaki-Keen A.E., Putkey F.R., Hjelmeland L.M. (1996). ARPE-19, a human retinal pigment epithelial cell line with differentiated properties. Exp. Eye Res..

[67] Samuel W., Jaworski C., Postnikova O.A., Kutty R.K., Duncan T., Tan L.X., Poliakov E., Lakkaraju A., Redmond T.M. (2017). Appropriately differentiated ARPE-19 cells regain phenotype and gene expression profiles similar to those of native RPE cells. Mol. Vis..

[68] Matsui M., Sakurai F., Elbashir S., Foster D.J., Manoharan M., Corey D.R. (2010). Activation of LDL receptor expression by small RNAs complementary to a noncoding transcript that overlaps the LDLR promoter. Chem. Biol..

