# Supplementary material for: Low-Density Lipoprotein Receptor (LDLR) Is Involved in Internalization of Lentiviral Particles Pseudotyped with SARS-CoV-2 Spike Protein in Ocular Cells
Source: Int J Mol Sci. 2023 Jul 24;24(14):11860. doi: 10.3390/ijms241411860 (PMC10380832; doi:10.3390/ijms241411860)
Supplement: Supplementary file 1 [file ijms-24-11860-s001.zip › ijms-2434214-supplementary.pdf]

Supplementary Figures and Tables

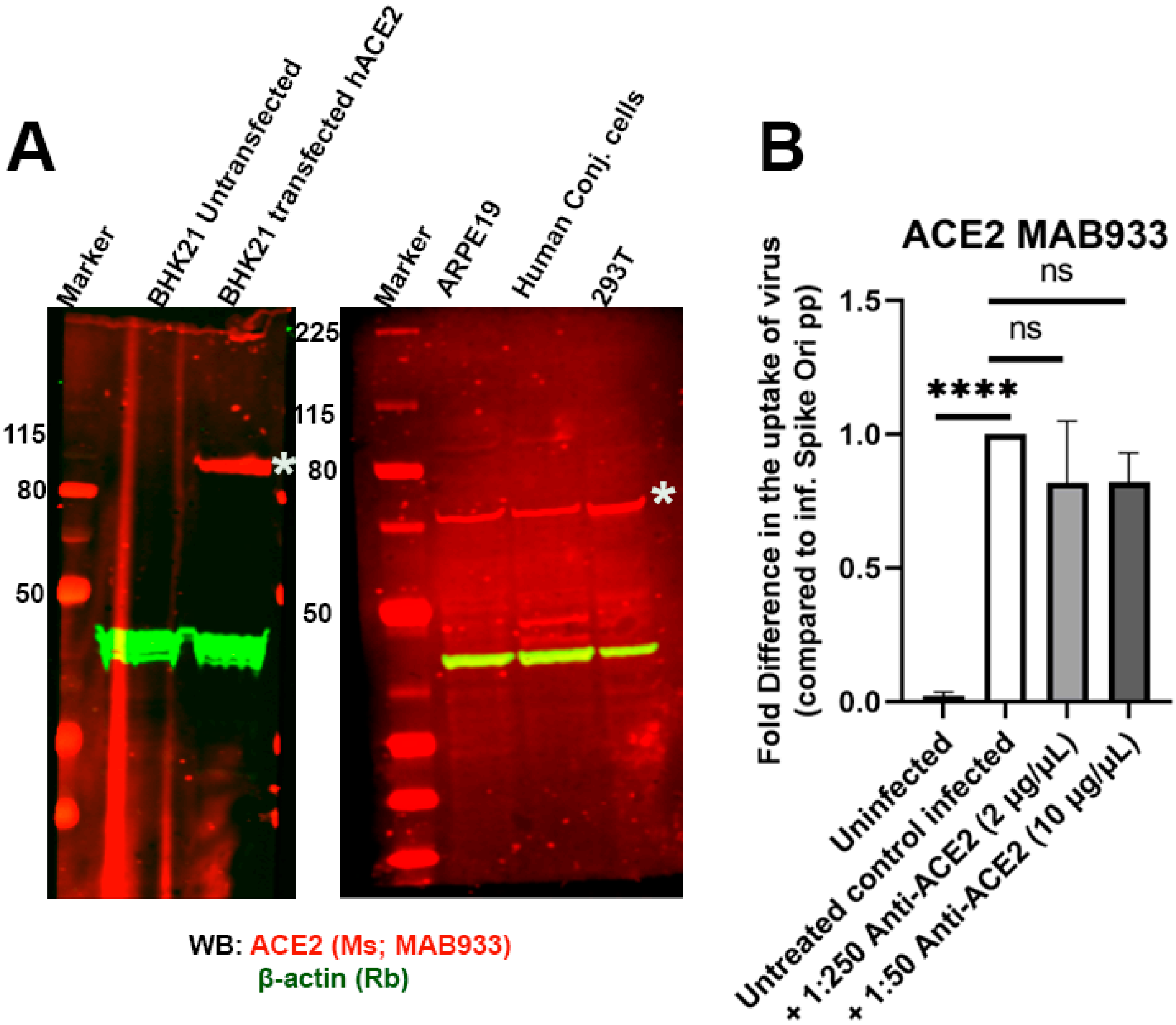

Figure S1. A. Expression of ACE2 (*; red) in different cell lines probed with anti-ACE2 antibodies (MAB933, 1:1000 dilution, 0.5 µg/mL). Expression of ACE2 in BHK21 cells untransfected or transfected with human ACE2 construct (left panel) and various other cell lines showing expression of ACE-2 protein (right panel). **B.** Blocking ACE2 does not affect pseudovirions uptake at MOI 10 in ARPE-19 cells. Fold difference in uptake of pseudovirions in the presence of 1:50 dilution (10 µg/mL) and 1:250 dilution (2 µg/mL) of MAB933 ACE2 antibody after 24 hours incubation. **** $p < 0.0001$.

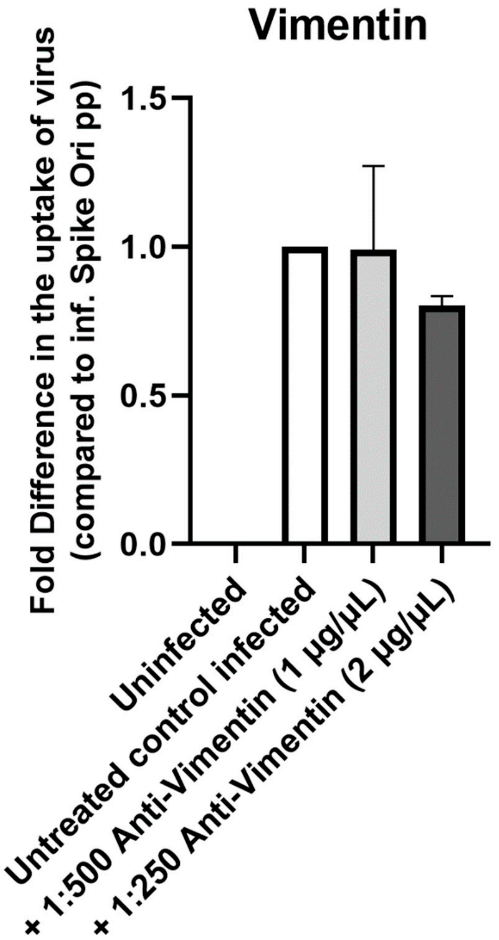

Figure S2. Antibody-mediated blocking of vimentin has no effect in blocking S protein pseudovirion infection of ARPE-19 cells. Fold difference in uptake of original spike-pseudotyped lentiviruses at MOI 10 in the presence of anti-vimentin antibody at 1:500 dilution (1 µg/mL) and 1:250 dilution (2 µg/mL) at 24 h compared to untreated controls infected with original spike protein pseudotyped particles alone.

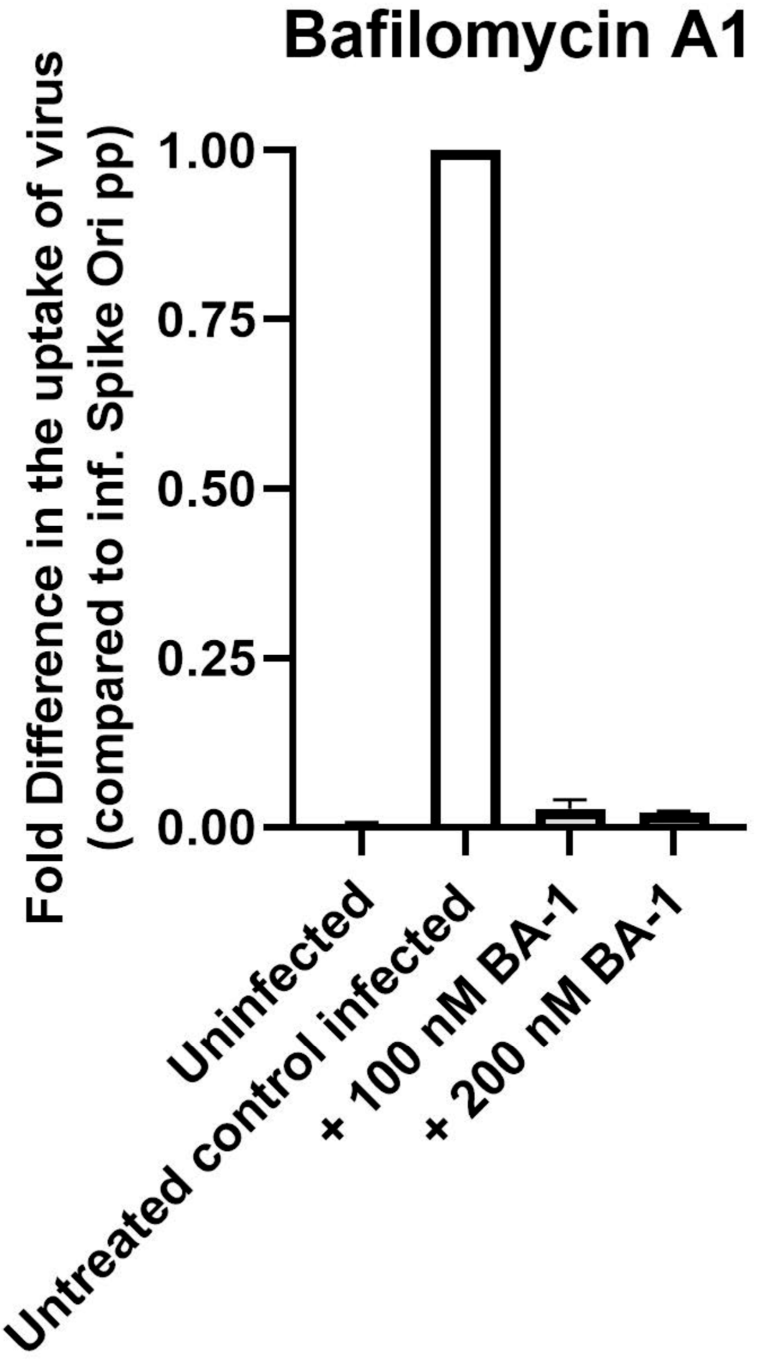

Figure S3. Bafilomycin A1, an agent for lysosomal alkalinization, inhibits S protein pseudovirion infection of ARPE-19 cells. Fold difference in uptake of original spike-pseudotyped lentiviruses at MOI 10 in the presence of 100 nM and 200 nM concentrations of bafilomycin at 48 h compared to untreated controls infected with original spike protein pseudotyped particles alone.

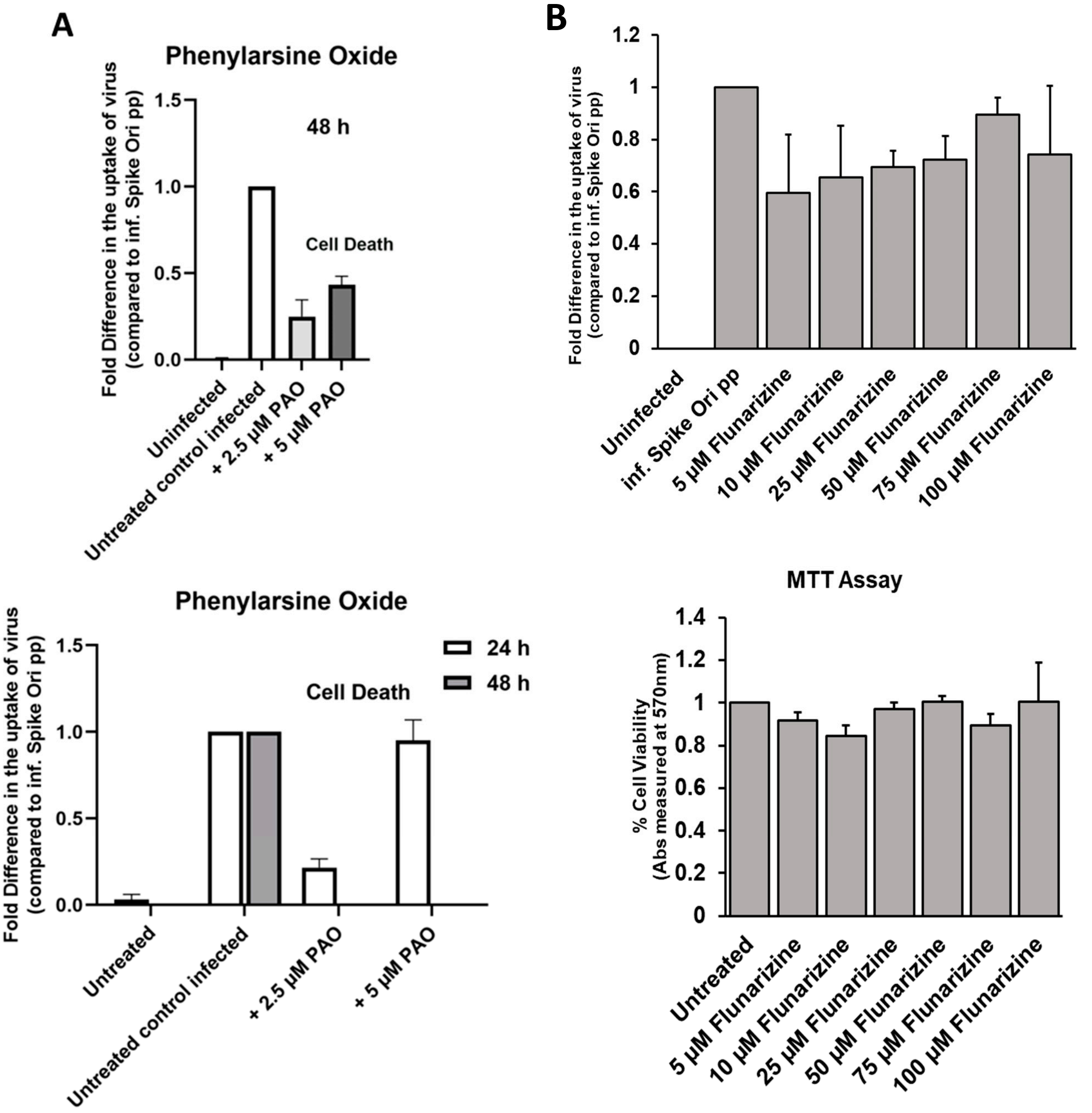

Figure S4. A. Effect of phenylarsine oxide (PAO), an inhibitor of clathrin-dependent endocytosis, in blocking S protein pseudovirion infection of ARPE-19 cells. Fold difference in uptake of original spike-pseudotyped lentiviruses at MOI 10 in the presence of 2.5 μM and 5 μM PAO at 48 h (top) and then repeated at 24 h and 48 h (bottom) compared to untreated controls infected with original spike protein pseudotyped particles alone. At the effective concentration of PAO, ARPE-19 cells showed significant cell death. **B.** Effect of flunarizine, T-type Ca^{2+} channel blocker and dynamin II GTPase activity inhibitor in blocking S protein pseudovirion infection of ARPE-19 cells at 48 h.

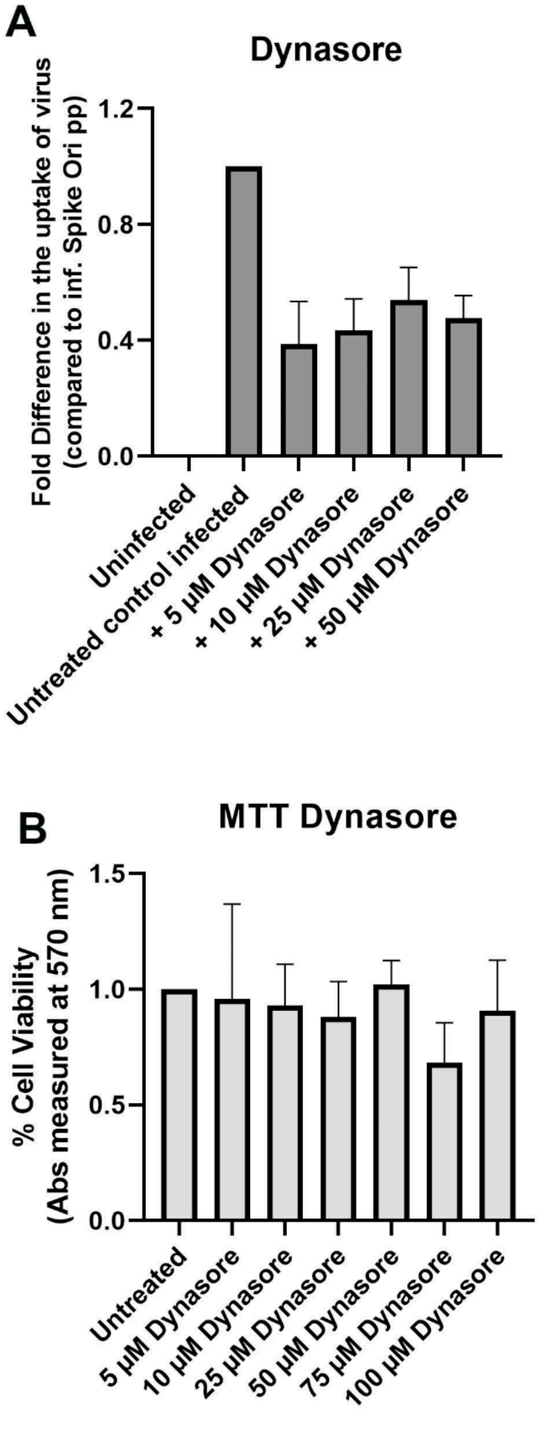

Figure S5. Dynasore, a noncompetitive inhibitor of dynamin GTPase activity, inhibits S protein pseudovirion infection of ARPE-19 cells. **A.** Fold difference in uptake of original spike-pseudotyped lentiviruses at MOI 10 in the presence of 5-50 µM concentrations of dynasore at 48 h compared to untreated controls infected with original spike protein pseudotyped particles alone. **B.** (%) cell viability under the same conditions measured by MTT cell viability assay.

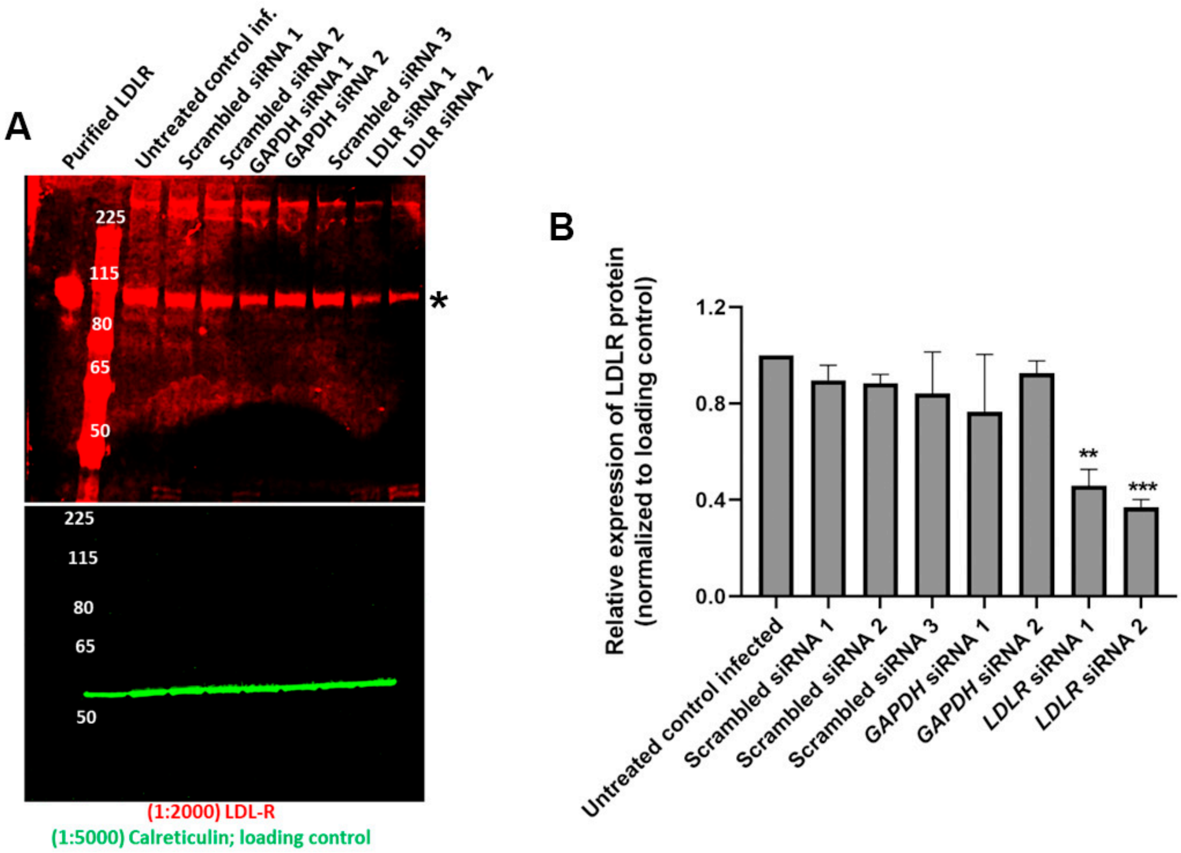

Figure S6. A. LDLR expression in ARPE-19 cells in the presence of various siRNAs.; immunoblot visualized with anti-LDLR monoclonal Abcam antibody (*; red) and Goat Anti-Calreticulin antibody (green). **B.** Relative expression of LDLR protein normalized to calreticulin loading control. ** $p < 0.001$, *** $p < 0.0005$.

Supplementary Table S1. LDLR and scrambled LDLR siRNA sequences.

Name	SiRNA sequence (Sense)
LDLR siRNA A	CGAAUUCCAGUGCUCUGAUtt
LDLR siRNA B	GGAGUGAACUGGUGUGAGAtt
Scrambled siRNA1	ACCTCGGTCTATTAAGCTGtt
Scrambled siRNA2	GAGAACGTGGTCGTATGAGtt
Scrambled siRNA3	ATTCGACTTGGTGCGATTCtt

Supplementary Table S2. A. Selection for each codon estimated using the HyPhy model, LDLR alignment. dN is the ratio of non-synonymous mutations, dS is the ratio of non-synonymous mutations. Positive dN-dS indicate an excess of dN over dS (suggesting positive selection). Small P values indicate support of positive selection.

Codon#	Codon Start	Triplet	dN-dS	*p*-Value
3	7	CCC	2.5	0.131687243
490	2353	AGT	3.234241776	0.146741991
26	76	AGA	2.500839556	0.14754001
183	547	CGC	2.331346502	0.186728073
163	487	CAG	2.134846117	0.282646669
498	2377	GTC	1.844830362	0.302946438
12	34	GTC	1.483332948	0.30639669
71	211	GGG	1.888477342	0.312174014
49	145	AGC	1.74042198	0.344458344
146	436	CTC	1.498741511	0.351534331
185	553	AGG	1.692795937	0.355655925
465	2068	CAC	1.745733131	0.356706709
182	544	CAG	1.737757155	0.359551108
328	1381	GGC	1.403153159	0.361980158
188	571	CAA	1.675603329	0.36719933
212	643	CGC	1.391657611	0.3833148
2	4	GGG	1.372496852	0.389630893
479	2116	AGG	1.398150694	0.391770761
154	460	CAG	1.569371806	0.40502763
451	2017	AGC	1.269379729	0.424378612
35	103	CAA	1.404910394	0.432915137
107	319	AAG	1.344783572	0.432973246
245	1120	GGC	1.310865417	0.443941435
110	328	TCC	1	0.444444444
264	1189	TCC	0.993085174	0.452754542
86	256	TTC	1.302584868	0.45297263
55	163	GGC	0.989648187	0.453790934
103	307	GGC	0.9870611	0.456172824
333	1402	GTC	0.98320435	0.459758639
59	175	TCC	0.975280769	0.467259522
440	1984	AGA	0.987981801	0.471626065
210	637	AGC	1.278621068	0.478381561
123	367	TCT	0.956390216	0.479634978
152	454	AGC	1.26588504	0.492966262

380	1543	AAC	1.119383508	0.497079141
202	613	CTA	0.767300201	0.508572081
85	253	CAG	1.136360429	0.513102956
234	1087	ACC	0.731484866	0.53311207
395	1711	CTC	0.5	0.592592593
325	1372	AGA	0.409812748	0.595005922
329	1384	GTC	0.574597924	0.596261587
11	31	ACC	0.481807262	0.59872978
124	370	CGG	0.343349201	0.607155736
7	19	AAA	0.533466235	0.608646227
200	607	CAC	0.86034016	0.609237817
349	1450	ATC	0.511829704	0.609314658
72	214	GAC	0.865478335	0.613557621
131	391	GAC	0.849979144	0.619191738
447	2005	AGG	0.415925537	0.621286406
174	520	GAA	0.270486826	0.639537402
317	1348	ATG	0.831448535	0.66037956
427	1807	AAG	0.333009273	0.663603945
511	2575	GTG	0.5	0.666666667
106	316	CCC	0.5	0.666666667
262	1171	GCC	0.5	0.666666667
266	1195	GCC	0.5	0.666666667
304	1309	GCC	0.5	0.666666667
321	1360	ACC	0.5	0.666666667
15	43	CTC	0.499065539	0.667914948
79	235	GTC	0.497093818	0.67056423
505	2557	GTC	0.497093818	0.67056423
496	2371	TCC	0.5	0.671341198
145	433	GTG	0.051765217	0.671375068
269	1204	TTC	0.294493693	0.671936728
9	25	CGC	0.497098136	0.672624192
485	2137	ACA	0.490110064	0.68011934
209	634	TCC	-0.196902413	0.684841877
360	1483	CTG	0.001189804	0.68716571
4	10	TGG	0.189827491	0.695795039
492	2359	GTG	0.474225442	0.70290057
339	1420	CAG	0.579127527	0.708149131
255	1150	CAG	0.574268244	0.714141273
192	583	AGC	-0.235916986	0.717587898
477	2107	CTG	0.079018473	0.719638195
396	1714	AGT	0.05453552	0.725009092
290	1267	ATC	0.457820443	0.728087482
14	40	TTG	0.055173855	0.728973539
251	1138	GAG	0.038750326	0.729571717
261	1168	AAG	0.018121811	0.734011861
207	628	ATC	0.453827103	0.734494108
13	37	GCC	0	0.740740741
19	55	GCG	0	0.740740741
196	595	GCC	0	0.740740741
249	1132	CAG	0.489115423	0.743538336
141	421	GCC	-0.019243806	0.746387601
149	445	GGT	-0.036632585	0.7513951
61	181	GAG	0.464287353	0.75334561

256	1153	CTG	-0.249757607	0.753719495
394	1708	CTC	-0.051826675	0.755696392
369	1510	AAG	0.447520906	0.75806164
252	1141	GAA	-0.417476805	0.758332077
216	655	GGC	-0.348833164	0.760446267
319	1354	TGC	0.498521147	0.760961791
371	1516	GTG	-0.077175359	0.762717873
190	577	GAC	-0.190625867	0.779581513
351	1456	AGC	-0.15378124	0.782765074
111	331	CAG	-0.20053611	0.784670906
151	451	GCC	-0.47596974	0.785755916
257	1156	GAC	0.423886289	0.788085608
25	73	GAC	0.42196168	0.789961148
305	1312	AGC	0.42196168	0.789961148
21	61	ACT	-0.5	0.790123457
486	2335	GGA	-0.553694411	0.798019484
323	1366	CTT	-0.365749497	0.829430789
132	394	CGG	-0.956675335	0.833529771
157	469	AGC	-1.339135037	0.833701942
426	1804	GAA	-0.991546442	0.838508284
197	598	TTC	-0.901123073	0.851264852
130	388	TCA	-0.81923379	0.852346522
117	349	CAC	-0.550229976	0.854658031
422	1792	ATC	-1.300190927	0.861328495
191	580	AGT	-1.257344237	0.869408504
259	1162	CAC	-0.668661983	0.877149236
115	343	CGC	-0.931907771	0.877732049
6	16	TGG	-1.390895034	0.880797618
41	121	TCC	-0.5	0.888888889
472	2089	GCC	-0.5	0.888888889
291	1270	CCC	-0.5	0.888888889
23	67	GTG	-1	0.888888889
105	313	CCC	-1	0.888888889
448	2008	ACC	-1	0.888888889
397	1717	GGC	-0.501029682	0.889071763
408	1750	TCC	-0.504281638	0.889597978
260	1165	ACG	-0.50469338	0.889719518
499	2380	CTC	-0.505728722	0.889901745
322	1363	CAG	-0.627582223	0.890666133
20	58	GGG	-0.516088827	0.891141925
493	2362	AGG	-1.021851872	0.891435626
67	199	ACC	-0.516077387	0.89170337
53	157	CAG	-1.532007158	0.895148827
204	619	GGC	-0.538925855	0.895556029
64	190	TTG	-0.835430789	0.895655424
494	2365	GCT	-1.061994371	0.895977511
142	424	TCC	-1.080818205	0.896220714
98	292	GGC	-0.550197054	0.89720447
50	148	GCT	-1.5	0.899862826
171	511	CCC	-1.5	0.899862826
189	574	GGG	-1.532941066	0.902021571
88	262	AGG	-0.588730563	0.902552533
144	430	CCG	-1.19694109	0.902642053

17	49	GCC	-1.541912746	0.903103408
93	277	GTG	-1.147170886	0.904913657
33	97	CAG	-0.745752016	0.905454195
60	178	CAG	-1.956899129	0.910966788
454	2032	CAG	-0.821204454	0.912085563
125	373	CAG	-0.826397915	0.91345864
463	2059	ATC	-0.782711365	0.927592728
437	1837	GTC	-0.789829297	0.928320584
308	1321	ATC	-0.797029356	0.929046906
28	82	GAA	-2.039883728	0.929113261
8	22	TTG	-1.255731199	0.929779002
294	1279	AGG	-0.83514948	0.931683857
353	1462	ATC	-0.838507379	0.933044124
378	1537	AGG	-1.544548537	0.933284264
438	1840	TTT	-0.864395708	0.93473023
383	1552	AAG	-0.916160552	0.938445804
113	337	GAG	-0.979105169	0.942044785
222	673	AAA	-0.969549848	0.9424574
187	559	CTT	-1.90301208	0.946435246
69	205	AAA	-1.848799971	0.949173842
413	1765	GAT	-1.780018692	0.949324969
338	1417	ATC	-1.295343356	0.950945246
488	2347	AAG	-1.911649146	0.951828863
96	286	GAC	-1.925594799	0.952697336
80	238	AAC	-1.14476937	0.954812734
74	220	AGC	-1.144799345	0.954814344
449	2011	ACC	-2.503045144	0.954855448
331	1396	GAC	-1.147063627	0.954935741
42	124	TAC	-2.141072161	0.955419284
238	1099	CTC	-1.369555451	0.955779929
231	1078	GAT	-2.135449858	0.962900526
127	379	GTC	-1.5	0.962962963
166	496	GCC	-1.5	0.962962963
402	1732	GTT	-1.5	0.962962963
159	475	ACC	-2.217826435	0.963784597
491	2356	AGC	-3.166987479	0.963823962
359	1480	GTC	-1.526528134	0.964245122
5	13	GGC	-1.570349437	0.966246014
385	1558	AGG	-1.638717883	0.968972161
336	1411	AGA	-3.0952209	0.970959092
476	2104	ATG	-5.481352291	0.972202063
102	304	CAA	-2.192968623	0.972538707
24	70	GGC	-1.927907782	0.976974819
99	295	TCA	-2.323443364	0.978416379
497	2374	ATT	-2.052798867	0.981192973
501	2386	ATC	-3.530866032	0.981294701
374	1525	AAA	-2.257123418	0.984326533
487	2338	AAT	-3.135496417	0.984703381
246	1123	TAC	-2.589036603	0.985660418
464	2065	CCC	-2.387691882	0.985798049
217	658	CCC	-2.5	0.987654321
169	505	AAC	-3.485314837	0.988993767
301	1300	ACG	-2.61554071	0.989270116

335	1408	AGC	-2.615985148	0.989483068
316	1345	AGA	-2.774390332	0.990674068
327	1378	CAC	-4.201758278	0.994290388
92	274	CAA	-5.023345529	0.997691932
362	1489	ACT	-5.2371476	0.99795646
428	1810	AGG	-4.666255436	0.998676975
412	1762	ATC	-5.524644679	0.999534393
284	1249	AGC	-6.280125546	0.999767947
376	1531	TTA	-3.228271408	1
387	1564	ATC	-3.755670631	1
469	2080	TTT	-2.97564776	1
46	136	TGC	-4.105175216	1
274	1219	CAC	-4.184902031	1
474	2098	GAC	-4.29981346	1
226	1063	ATC	-4.841066263	1
201	610	TGC	-5.366414017	1
243	1114	GAG	-5.627099809	1
507	2563	CTG	-0.616491883	1
293	1276	CTG	-0.61688089	1
242	1111	CTG	-0.627143599	1
299	1294	CTG	-0.64035991	1
450	2014	CTG	-0.858110461	1
16	46	CTC	-0.935896037	1
268	1201	CTC	-0.935896037	1
289	1264	CTC	-0.935896037	1
399	1723	CTC	-0.978747302	1
81	241	CGC	-0.982574505	1
22	64	GCA	-1	1
37	109	GGG	-1	1
66	196	GTC	-1	1
363	1492	GTC	-1	1
368	1507	ACC	-1	1
398	1720	CGC	-1	1
442	1990	GTG	-1	1
500	2383	CCC	-1	1
65	193	TCT	-1	1
137	409	GGC	-1	1
193	586	CCC	-1	1
332	1399	ACC	-1	1
366	1501	GCG	-1	1
382	1549	TCC	-1	1
386	1561	GCC	-1	1
404	1738	TCC	-1	1
417	1777	GGC	-1	1
441	1987	GGA	-1	1
373	1522	AGG	-1.067321451	1
281	1240	CTG	-1.228808355	1
122	364	ATC	-1.256505389	1
318	1351	ATC	-1.256967857	1
334	1405	ATC	-1.256967857	1
409	1753	ATC	-1.259465686	1
164	490	CTG	-1.28071982	1
213	646	TGT	-1.308503021	1

90	268	GAT	-1.31147567	1
393	1582	CAT	-1.31147567	1
54	160	GAT	-1.321128009	1
178	532	GAT	-1.321128009	1
250	1135	TGT	-1.333475292	1
509	2569	GAT	-1.333475292	1
423	1795	TTG	-1.353386437	1
435	1831	TTG	-1.357489297	1
38	112	AAA	-1.400377501	1
405	1741	AAA	-1.400377501	1
315	1342	CAG	-1.411559767	1
101	301	GAG	-1.415993367	1
420	1786	AAG	-1.415993367	1
468	2077	AAG	-1.415993367	1
247	1126	AAG	-1.416404896	1
508	2566	GAG	-1.416404896	1
225	682	GAG	-1.416981895	1
285	1252	GAG	-1.416981895	1
462	2056	CAG	-1.417102697	1
179	535	GAG	-1.417398971	1
278	1231	AAG	-1.418028105	1
114	340	TTT	-1.502880723	1
506	2560	AGT	-1.502880723	1
455	2035	TAT	-1.534950878	1
68	202	TGC	-1.551042488	1
248	1129	TGC	-1.551042488	1
267	1198	TAC	-1.551042488	1
288	1261	AGC	-1.551042488	1
357	1474	GAC	-1.551042488	1
400	1726	TAC	-1.551042488	1
418	1780	AAC	-1.551042488	1
116	346	TGC	-1.558108178	1
160	478	TGC	-1.568089016	1
233	1084	GAC	-1.568089016	1
236	1093	AGC	-1.568089016	1
443	1993	AAC	-1.568089016	1
109	325	TGC	-1.568116955	1
199	604	TTC	-1.568116955	1
218	661	GAC	-1.568116955	1
168	502	GAC	-1.570227681	1
254	1147	TTC	-1.570227681	1
342	1429	GAC	-1.570227681	1
377	1534	TTC	-1.570227681	1
411	1759	AGC	-1.570227681	1
30	88	AAC	-1.575426958	1
172	514	GAC	-1.575426958	1
309	1324	TAC	-1.584535202	1
406	1744	CTT	-1.896114084	1
48	142	GGC	-2	1
76	226	GGG	-2	1
78	232	CGT	-2	1
84	250	CCT	-2	1
147	439	ACC	-2	1

158	472	TCC	-2	1
162	484	CCC	-2	1
186	556	GGT	-2	1
258	1159	CCC	-2	1
287	1258	ACC	-2	1
296	1285	GTG	-2	1
298	1291	GCT	-2	1
330	1387	TCT	-2	1
346	1441	GTG	-2	1
365	1498	GTT	-2	1
375	1528	ACG	-2	1
381	1546	GGC	-2	1
388	1567	GTG	-2	1
392	1579	GTT	-2	1
410	1756	TCA	-2	1
452	2023	GGC	-2	1
466	2071	TCG	-2	1
481	2125	AGG	-2.080926841	1
307	1318	AGA	-2.099118295	1
83	247	ATT	-2.468207438	1
104	310	TGT	-2.638982544	1
135	403	TTG	-2.648097424	1
95	283	TGC	-2.678568239	1
128	382	TGT	-2.683652834	1
239	1102	TGC	-2.734206045	1
457	2041	TGC	-2.745178534	1
458	2044	CTC	-2.759903913	1
139	415	GAC	-2.78717825	1
283	1246	CGG	-2.788170132	1
273	1216	CGG	-2.814822551	1
228	1069	GAG	-2.832067329	1
58	172	GAG	-2.832655784	1
120	358	AAG	-2.832685788	1
198	601	GAG	-2.833391405	1
89	265	TGC	-2.857198345	1
148	442	TGT	-2.872126704	1
97	289	AAC	-2.882461203	1
227	1066	GAT	-2.939567203	1
214	649	GAT	-2.94073694	1
47	139	GAT	-2.941176867	1
18	52	GCG	-3	1
91	271	GGC	-3	1
150	448	CCC	-3	1
297	1288	GTC	-3	1
341	1426	CCC	-3	1
384	1555	CCA	-3	1
45	133	GTC	-3	1
108	322	ACG	-3	1
119	355	GGG	-3	1
223	676	TCT	-3	1
311	1330	TCT	-3	1
356	1471	ACC	-3	1
358	1477	TCT	-3	1

391	1576	CCT	-3	1
430	1816	GCC	-3	1
489	2350	CCC	-3	1
306	1315	AAT	-3.002562287	1
203	616	AGT	-3.011921488	1
129	385	GAC	-3.065986255	1
206	625	TGC	-3.065986255	1
126	376	TTC	-3.070020593	1
224	679	GAC	-3.070020593	1
170	508	GAC	-3.095551005	1
134	400	TGC	-3.099286194	1
156	466	AAC	-3.103463418	1
272	1213	AAC	-3.103463418	1
350	1453	CAC	-3.103463418	1
324	1369	GAC	-3.117778794	1
155	463	TGC	-3.135613763	1
29	85	AGA	-3.17983137	1
62	184	ACG	-4	1
370	1513	GGC	-4	1
56	166	TCT	-4	1
181	541	CCG	-4	1
244	1117	GGT	-4	1
276	1225	GTC	-4	1
345	1438	GCT	-4	1
364	1495	TCT	-4	1
416	1774	GGG	-4	1
473	2095	CCG	-4	1
512	2578	GCG	-4	1
75	223	TGT	-4.011096061	1
118	352	GAT	-4.027221121	1
27	79	TGC	-4.043610332	1
367	1504	GAT	-4.105979324	1
184	550	TGT	-4.106043176	1
390	1573	GAT	-4.106043176	1
167	499	TGC	-4.129755135	1
112	334	GAC	-4.144382406	1
510	2572	GAC	-4.178953734	1
471	2086	TGC	-4.184902031	1
34	100	TGC	-4.2012941	1
208	631	CAC	-4.545998768	1
136	406	GAC	-4.551911561	1
133	397	GAC	-4.572519446	1
425	1801	GAT	-4.58314455	1
403	1735	GAC	-4.5893252	1
173	517	TGC	-4.594057155	1
295	1282	AAC	-4.617897604	1
36	106	GAC	-4.645385468	1
100	298	GAC	-4.666878067	1
277	1228	AGG	-4.734554576	1
232	1081	CCC	-5	1
421	1789	ACC	-5	1
70	208	TCC	-5	1
177	529	TCG	-5	1

195	592	TCG	-5	1
280	1237	ACG	-5	1
461	2053	CCG	-5	1
175	523	GAT	-5.406193745	1
415	1771	AAC	-5.445799211	1
431	1819	CAC	-5.674315628	1
138	412	TCA	-6	1
215	652	GGT	-6	1
459	2047	CCT	-6	1
265	1192	ATC	-6.731512457	1

Supplementary Table S2. B. Detection of positions in the LDLR sequence alignment that are likely to experience episodic positive selection (the MEME program).

Position	*p*-Value
252	<0.01
769	<0.01
26	0.05
397	0.04
678	0.04
754	0.04
764	0.04
107	0.03
727	0.03
746	0.01

Supplementary Table S3. A. Selection for each codon estimated using the HyPhy model, ACE2 alignment. dN is the ratio of non-synonymous mutations, dS is the ratio of non-synonymous mutations. Positive dN-dS indicate an excess of dN over dS (suggesting positive selection). Small P values indicate support of positiove selection

Codon#	Codon Start	Triplet	dN-dS	*p*-Value
757	2356	ATT	4.15433764	0.054315214
91	277	GTC	3.406238154	0.070780305
628	1969	AAA	4.05117411	0.093565959
601	1888	GAT	3.588988964	0.095854024
646	2023	TTG	2.969974358	0.106243179
660	2065	AAA	3.698822463	0.123053682
274	856	GGA	2.517423449	0.15458334
465	1471	GTG	2.113408065	0.159944441
602	1891	AAA	3.180990445	0.163912618
627	1966	TTA	2.039198042	0.164287258
65	199	GAC	2.357206635	0.182108744
216	682	CAT	2.746756762	0.1867426
764	2377	CCA	2.037979435	0.189899843
154	466	TTA	2.065937871	0.190460819
747	2326	AGT	2.30749999	0.199782388
730	2275	ATC	2.327056105	0.201175002
711	2218	TCC	1.970569188	0.209598512
39	121	TAT	2.42067825	0.214069182
738	2299	GAT	2.238692957	0.231821888

696	2173	CTG	1.380747099	0.23312519
148	448	GAA	2.272009313	0.234303954
62	190	AAT	2.239857845	0.234515195
744	2317	AAA	2.641671645	0.234864665
219	691	GAA	1.901941501	0.25869672
767	2386	CAA	2.300928885	0.26087691
629	1972	GTA	2.347277499	0.261566693
687	2146	CGT	2.175426498	0.274796816
507	1606	GAA	2.240066529	0.283617474
57	175	GTC	2.132350333	0.283778488
539	1702	CTA	1.691507174	0.28786219
314	976	GGA	1.56771926	0.29353042
726	2263	GTT	1.498059514	0.297449196
617	1936	TCT	1.492003459	0.301805115
12	34	GTT	1.490051015	0.302271073
394	1258	TCC	1.491976012	0.302727762
331	1027	GTC	1.480912332	0.307901604
63	193	GCT	1.48050854	0.30910477
183	553	GTC	1.470746622	0.314330426
206	652	AGC	2.108826966	0.322110663
243	763	TAT	2.010665893	0.323479977
361	1159	GCA	1.709133157	0.342514141
758	2359	AGC	1.969385674	0.347390753
286	892	GTG	1.424441676	0.349090964
113	343	CGG	1.413690466	0.350084324
3	7	AGC	1.412431653	0.354893198
634	1987	ATT	1.411640652	0.355490116
680	2125	AGC	1.723465742	0.35821546
77	235	CTT	1.592184063	0.366628759
89	271	CTC	1.507069152	0.375634232
200	634	GTA	1.404949485	0.385877571
220	694	GAG	1.574754018	0.405939509
684	2137	GAT	1.340857769	0.414812526
41	127	AGT	1.340666916	0.414989705
325	1009	GGA	1.324543738	0.415071029
84	256	CAA	1.579076234	0.420452258
624	1957	CAG	1.707266758	0.423080714
61	187	AAT	1.330884355	0.424208182
551	1738	AAT	1.330884355	0.424208182
543	1714	AAT	1.335973122	0.424325909
723	2254	GTG	0.965325262	0.428866352
82	250	CCA	1.028093145	0.436236029
760	2365	GGA	1.050701389	0.436990228
722	2251	GGA	1.101862803	0.43911134
168	508	TCT	1	0.444444444
716	2233	GTT	0.995967964	0.44805027
158	478	GAG	1.344315163	0.45017226
547	1726	GCA	0.993765349	0.451908777
759	2362	AAA	1.365371465	0.453682983
341	1057	AAG	1.358688514	0.456363298
105	319	GTG	1	0.46090535
635	1990	CTT	1	0.46090535
239	751	GCC	1	0.46090535

745	2320	GCA	0.98611438	0.461328583
289	901	GCC	0.967595967	0.471570898
120	364	ACA	0.975013681	0.471765641
96	292	CAG	1.269773131	0.471994286
710	2215	GTT	0.958695594	0.474498444
733	2284	TTC	1.281237931	0.475456335
772	2401	GTT	0.94860267	0.47781653
503	1594	GCA	0.926329543	0.48595246
669	2092	ACT	0.912584048	0.489636431
676	2113	AGG	0.947652437	0.49490212
28	88	GAC	1.275447373	0.496403548
403	1285	CAA	1.19484481	0.497869613
5	13	TCC	0.941690358	0.501188659
755	2350	ATC	0.867313458	0.508161641
746	2323	AGA	0.860010309	0.522238042
293	913	CAG	0.976002428	0.527619683
707	2206	CAG	1.120212789	0.529651981
141	427	TTA	0.634549632	0.529682725
58	178	CAA	1.118464402	0.533219122
553	1744	AGG	0.799968581	0.535020798
313	973	CAA	1.117073764	0.54291354
587	1846	CAA	1.114028908	0.545188478
134	406	GAT	0.850759441	0.545542986
563	1774	TTT	0.899313335	0.549535073
548	1729	AAG	0.922984624	0.556462086
101	307	AAT	0.892138545	0.560912276
615	1930	CGA	0.45803496	0.562814447
401	1279	GAT	0.888612073	0.562850509
638	1999	GAG	0.919013801	0.563282139
689	2152	AAT	0.887256237	0.564572032
743	2314	AAT	0.887256237	0.564572032
283	883	GAT	0.888119363	0.565816748
317	985	GAA	0.801975003	0.567330351
457	1447	GAG	0.903692927	0.588823723
699	2182	CAG	0.732471934	0.588870921
685	2140	GCT	0.5	0.592592593
196	622	GAA	0.893543498	0.599194111
247	775	ATT	0.480404279	0.59920109
663	2074	TCT	0.480198609	0.599504902
170	514	GTC	0.47630769	0.600575617
112	340	AAA	0.90794061	0.602057414
85	259	GAA	0.893816469	0.602663037
692	2161	AGC	0.858031162	0.603686355
748	2329	GGA	0.891881775	0.606607661
703	2194	GGA	0.141801213	0.611207239
753	2344	GCC	0.444195702	0.611398464
328	1018	CAG	0.568856079	0.614218748
568	1789	GAC	0.578342163	0.618100767
107	325	TCA	0.616564067	0.61859596
104	316	TCA	0.613734874	0.619680479
132	400	AAC	0.838821268	0.636114832
152	460	AAC	0.243769848	0.640693138
76	232	ACA	0.408838787	0.646872815

580	1825	GAC	0.445585908	0.649089008
38	118	TTC	0.314773154	0.653088052
527	1666	AAT	0.373382591	0.654160603
204	646	GAC	0.159715256	0.654985403
653	2044	AAT	0.354702095	0.657549146
765	2380	GGA	0.527107271	0.658849418
128	388	GGA	0.52521209	0.661226817
29	91	AAG	0.348752901	0.661500779
470	1486	ACA	0.506782351	0.663665857
25	79	ACA	0.5	0.666666667
45	139	TCT	0.5	0.666666667
386	1234	GCA	0.5	0.666666667
476	1504	TCT	0.5	0.666666667
481	1519	TCT	0.5	0.666666667
598	1879	GCT	0.5	0.666666667
15	43	ACT	0.499833896	0.666888213
127	385	ACT	0.499829548	0.666894014
202	640	GGC	0.249282226	0.666960448
69	211	GCC	0.499413461	0.667449638
673	2104	AAG	0.31791651	0.669125418
217	685	ACC	0.498021003	0.669315815
531	1678	CTT	0.495756979	0.672372447
642	2011	CGA	0.035020404	0.674360531
208	658	GGC	0.035554691	0.676298159
83	253	CTA	0.018751343	0.682207993
491	1558	CTT	0.47870449	0.696323808
42	130	TCA	0.153566829	0.696469707
578	1819	AGT	0.184201321	0.698185322
111	337	AGC	0.1699051	0.701429991
769	2392	ACT	-0.119368435	0.701838281
108	328	GAA	0.479220158	0.705677112
211	667	ATT	0.468997152	0.71073637
588	1849	AGC	0.06780063	0.7235989
275	859	CAG	0.56660479	0.723916668
662	2071	GTG	0.045915677	0.726826259
502	1591	CAA	0.57146057	0.729917107
133	403	CCA	0.023570824	0.733784508
79	241	CAA	0.561758632	0.736727107
8	22	CTT	0.009246466	0.737988399
40	124	CAA	0.556889752	0.738412046
574	1807	TTT	0.038782965	0.738863693
23	73	GCC	0	0.740740741
97	295	GCT	0	0.740740741
322	1000	ACG	-0.002803152	0.74157014
618	1939	GTT	-0.008072081	0.743122804
116	352	ACA	-0.014152344	0.744904297
234	736	GCA	-0.528435653	0.745738717
310	964	AAT	-0.033133863	0.746006805
64	196	GGG	-0.020101304	0.746619794
763	2374	AAT	0.446069273	0.74726809
24	76	AAG	-0.042667858	0.747748211
327	1015	GTT	-0.02440145	0.747882298
655	2050	TTT	0.446221309	0.750289699

165	499	AGC	-0.034995308	0.750927469
273	853	TTT	0.443916395	0.751258714
47	145	AAT	0.443628118	0.751380085
291	907	GAT	0.443628118	0.751380085
123	373	ACC	-0.040708889	0.752555862
564	1777	ACC	-0.042435108	0.753045915
741	2308	AAG	-0.034038321	0.758909409
406	1294	AAT	-0.461732301	0.758956221
169	511	GAG	0.452391803	0.76797742
390	1246	AAG	0.454052866	0.768032374
445	1411	GAC	-0.145963641	0.769867516
157	475	AAT	-0.544552969	0.771197019
143	433	GAA	-0.162807183	0.772837665
329	1021	AAA	0.451494067	0.773897566
533	1684	AAA	0.448869943	0.775796345
449	1423	AAA	0.454158098	0.775851672
393	1255	AAA	0.453868737	0.77596249
129	391	AAA	0.449823706	0.776317512
33	103	GAA	0.452308798	0.776560451
742	2311	AAA	0.449038664	0.776608627
630	1975	AAA	0.449589755	0.777604931
444	1408	AAA	0.44926958	0.777728107
530	1675	AGG	-0.598806329	0.778407434
520	1645	GAA	0.44599663	0.778989494
542	1711	GAA	0.445941605	0.779010735
658	2059	GCA	-0.450005312	0.779153335
567	1786	AAA	-0.216419233	0.782722527
66	202	AAA	0.445040516	0.783172254
294	916	AGA	-0.199109897	0.792365228
766	2383	TTC	0.420502255	0.792702844
197	625	GTA	-0.534958667	0.796161906
677	2116	ATG	2.078107729	0.797905806
650	2035	ATC	-0.229182955	0.800803953
284	886	GCA	-0.631256149	0.811913816
571	1798	AAG	-0.68091727	0.8135399
632	1981	CAG	-0.367770654	0.816046878
631	1978	AAT	-0.907399585	0.816636053
644	2017	GCT	-0.818308991	0.821764987
700	2185	CCA	-0.994068622	0.825690229
724	2257	ATA	-0.727534839	0.827385619
754	2347	TCC	-1.071548436	0.834891147
201	637	GAT	-0.929377197	0.835403508
368	1180	AAT	-0.957244938	0.83864566
740	2305	AAG	-0.449455405	0.842009592
88	268	AAT	-0.435694731	0.842231053
762	2371	AAT	-0.99508149	0.842936388
227	715	CAT	-0.468417858	0.846237618
439	1393	AAA	-1.015372758	0.852850312
297	925	AAG	-0.550300809	0.856197788
147	445	AAT	-0.52198862	0.856301226
326	1012	AAT	-0.526469639	0.857154263
640	2005	GAT	-0.526469639	0.857154263
706	2203	AAC	-0.599701351	0.857608371

512	1621	AAA	-0.596030275	0.86349027
612	1921	TAC	-0.672842946	0.86674366
59	181	AAC	-0.635978299	0.869132806
529	1672	CTG	-0.643377358	0.870268412
226	712	GAA	-0.665765232	0.872678365
100	304	CAA	-1.352695859	0.875065332
93	283	CTT	-0.913958364	0.877627695
36	112	GAC	-0.711486586	0.881511878
613	1924	CTG	-0.725212354	0.881725437
399	1273	TCA	-0.476169459	0.881829715
193	613	GGA	-0.478322037	0.882467092
557	1756	AAC	-0.737845222	0.885576384
637	1996	GGG	-1.3620232	0.886119985
268	838	TCT	-0.9902612	0.886962093
305	949	TCT	-0.499053019	0.888608302
585	1840	GCA	-1	0.888888889
16	46	GCT	-0.5	0.888888889
78	238	GCC	-0.5	0.888888889
130	394	GTT	-0.5	0.888888889
230	724	GCC	-0.5	0.888888889
270	844	ACA	-0.5	0.888888889
725	2260	GTG	-0.5	0.888888889
727	2266	GGC	-0.5	0.888888889
734	2287	ACT	-0.5	0.888888889
68	208	TCT	-0.502864109	0.889396666
50	154	ACC	-0.50834821	0.890361176
22	70	CAG	-1.988050391	0.892713255
608	1909	GAC	-0.802559508	0.892783842
575	1810	GTG	-0.526423524	0.893228005
199	631	GGG	-0.536735193	0.893316251
526	1663	TTC	-1.070762208	0.896939245
80	244	ATG	-1.751781198	0.900783749
517	1636	AAC	-1.402287348	0.902141237
400	1276	CCC	-1.897893932	0.904800203
728	2269	ATT	-0.642890843	0.911082413
194	616	GAC	-1.538860977	0.91231051
737	2296	AGA	-0.685061304	0.912671544
67	205	TGG	1.200004926	0.913612158
605	1900	GAA	-2.305082118	0.913822419
151	457	GCA	-1.256092432	0.913872584
668	2089	AGA	-0.716604584	0.916835296
32	100	CAC	-2.748873439	0.920372714
732	2281	ATC	-0.727846665	0.921638136
124	376	ATC	-0.744778567	0.923542924
71	217	TTA	-1.475283855	0.923862533
87	265	CAG	-1.720445266	0.926454463
222	700	AAA	-1.760872159	0.935358729
304	946	GTA	-2.415085622	0.936216236
118	358	CTA	-1.343012992	0.937856486
506	1603	CAT	-0.908447245	0.938679169
645	2020	AAT	-0.911966327	0.939406386
770	2395	GAT	-0.912880746	0.939453615
771	2398	GAT	-0.931030473	0.940712462

81	247	TAT	-1.760159191	0.941355386
172	520	AAG	-1.010785908	0.94351837
643	2014	GTG	-1.865968153	0.944797242
55	169	GAG	-1.053921114	0.946827352
647	2026	AAA	-1.100816847	0.949354562
21	67	GAA	-1.113925437	0.950590819
212	670	GAA	-1.11531686	0.950672223
535	1690	GAA	-1.11952509	0.950717816
670	2095	GAA	-1.129760324	0.951578198
708	2209	CCC	-2.479440543	0.952961741
54	166	GAA	-1.150215676	0.953138559
709	2212	CCT	-2.5	0.95473251
13	37	GCT	-2	0.95473251
318	988	AAT	-1.147146132	0.954886418
691	2158	AAC	-1.146627883	0.954912415
768	2389	AAC	-1.152495101	0.95522507
301	937	AAG	-2.02384556	0.956761672
296	922	TTC	-1.192153638	0.957260034
519	1642	ACA	-2.572424427	0.957561842
560	1765	GAG	-2.093228335	0.957970397
115	349	AAC	-1.214513371	0.958349896
729	2272	GTC	-2.114844723	0.959788855
418	1330	CTC	-1.445056354	0.959954776
287	895	GAC	-3.08409911	0.960435343
505	1600	AAA	-2.186429582	0.961236956
145	439	GGT	-1.503038776	0.962903748
34	106	GCC	-1.5	0.962962963
136	412	CCA	-1.5	0.962962963
704	2197	CCT	-1.5	0.962962963
306	952	GTT	-1.501452439	0.963034604
72	220	AAG	-2.258172344	0.964206673
307	955	GGT	-1.544114674	0.965065189
119	361	AAT	-2.861195305	0.96769058
712	2221	ATA	-3.037666227	0.969425367
664	2077	GAT	-2.389914656	0.970001286
221	697	ATT	-1.807910016	0.974970252
450	1426	AAG	-2.995323816	0.976604813
633	1984	ATG	-3.769188114	0.976934255
244	766	ATC	-1.88734346	0.977241675
665	2080	ATC	-1.890251582	0.977319785
448	1420	ATG	0.340988981	0.977548696
342	1060	GGC	-4.681723029	0.981728893
320	994	ATG	0.339162611	0.982812735
719	2242	GTT	-3.138271118	0.984506392
245	769	AGT	-2.299231453	0.985592474
135	409	AAT	-3.239433909	0.985796458
607	1906	AAC	-3.238377078	0.986000996
549	1732	AAC	-3.252748967	0.986171575
14	40	GTA	-2.422164183	0.986401083
639	2002	GAG	-2.521760261	0.987558754
162	490	GCT	-2.5	0.987654321
330	1024	GCA	-2.5	0.987654321
509	1612	CCT	-2.5	0.987654321

603	1894	GCA	-2.5	0.987654321
419	1333	ACG	-2.504426442	0.987721361
678	2119	TCC	-2.5443272	0.987869101
207	655	CGC	-2.550074147	0.988249463
90	274	ACA	-2.574744202	0.988730237
198	628	AAT	-3.62096258	0.988828524
749	2332	GAA	-2.669224231	0.988945953
364	1168	TTT	-2.672198299	0.989854406
138	418	GAA	-3.785923902	0.990055998
311	967	ATG	0.335281687	0.994188905
137	415	CAA	-3.946011308	0.994396492
497	1576	CAA	-4.110840631	0.994895282
544	1717	GTT	-3.525424427	0.996005748
238	748	AAT	-3.692701181	0.996570975
622	1951	ATG	0.334435503	0.996704387
323	1003	GAC	-4.045957245	0.99717066
237	745	ATG	0.667561602	0.99732059
721	2248	ATG	0.334020217	0.997943586
474	1498	CCC	-5.5	0.999542753
541	1708	TTG	-2.090750441	1
487	1537	ATT	-2.350923029	1
102	310	GGG	-4	1
714	2227	CTG	-1.2556022	1
160	484	CTC	-1.921991027	1
589	1852	ATC	-2.376355737	1
248	778	GGA	-3	1
343	1063	GAC	-3.241795176	1
453	1435	GAG	-4.560673552	1
174	526	CTG	-0.62398535	1
321	997	CTA	-0.62398535	1
339	1051	CTG	-0.62398535	1
413	1315	CTG	-0.62398535	1
365	1171	CTG	-0.6278011	1
688	2149	CTG	-0.6278011	1
477	1507	CTG	-0.628979124	1
266	832	CTG	-0.642169286	1
210	664	TTG	-0.702527382	1
269	841	TTG	-0.702527382	1
236	742	TTG	-0.702809227	1
146	442	TTG	-0.707461657	1
27	85	TTG	-0.708482504	1
430	1366	TTA	-0.722408284	1
177	535	TTA	-0.722428799	1
140	424	TTA	-0.723510367	1
456	1444	CGA	-0.810590405	1
228	718	CTT	-0.960995513	1
142	430	CTT	-0.971580936	1
536	1693	CCC	-1	1
720	2245	GTG	-1	1
2	4	TCA	-1	1
19	55	TCC	-1	1
103	313	TCT	-1	1
214	676	GTG	-1	1

223	703	CCA	-1	1
241	757	CCT	-1	1
250	784	CTC	-1	1
251	787	CCT	-1	1
252	790	GCT	-1	1
256	802	GGT	-1	1
260	814	GGT	-1	1
264	826	ACA	-1	1
309	961	CCT	-1	1
319	991	TCC	-1	1
324	1006	CCA	-1	1
334	1036	CCC	-1	1
335	1039	ACA	-1	1
336	1042	GCT	-1	1
340	1054	GGG	-1	1
345	1111	ACA	-1	1
346	1114	GCT	-1	1
363	1165	CCT	-1	1
373	1195	GGA	-1	1
383	1225	TCA	-1	1
384	1228	CTT	-1	1
397	1267	CTT	-1	1
408	1300	ACA	-1	1
417	1327	GCA	-1	1
421	1339	GTT	-1	1
422	1342	GGG	-1	1
443	1405	CCC	-1	1
459	1453	GTT	-1	1
466	1474	CCC	-1	1
485	1531	TCA	-1	1
516	1633	TCA	-1	1
521	1648	GCT	-1	1
522	1651	GGA	-1	1
538	1699	ACC	-1	1
545	1720	GTA	-1	1
554	1747	CCA	-1	1
556	1753	CTC	-1	1
597	1876	TCA	-1	1
619	1942	GCA	-1	1
648	2029	CCA	-1	1
656	2053	GTC	-1	1
657	2056	ACT	-1	1
674	2107	GCC	-1	1
697	2176	GGG	-1	1
751	2338	CCT	-1	1
434	1378	AGG	-1.120920929	1
52	160	ATT	-1.130526226	1
666	2083	ATT	-1.130652435	1
117	355	ATT	-1.132955784	1
86	262	ATT	-1.134793396	1
736	2293	ATC	-1.231660104	1
595	1870	CTA	-1.259697216	1
525	1660	CTG	-1.26235091	1

698	2179	ATA	-1.306535168	1
501	1588	TGT	-1.341993485	1
438	1390	TTT	-1.342512464	1
695	2170	TTT	-1.342512464	1
225	709	TAT	-1.345648619	1
472	1492	TGT	-1.351271918	1
584	1837	TAT	-1.351271918	1
229	721	CAT	-1.353352136	1
479	1513	CAT	-1.353352136	1
70	214	TTT	-1.354145459	1
347	1117	CAT	-1.354145459	1
375	1201	CAT	-1.354145459	1
661	2068	AAT	-1.354145459	1
181	547	TAT	-1.358306695	1
752	2341	TAT	-1.358306695	1
186	562	AAT	-1.359050898	1
559	1762	TTT	-1.359050898	1
625	1960	TAC	-1.371883788	1
114	346	TTG	-1.372271866	1
184	556	TTG	-1.384392148	1
205	649	TAC	-1.402405874	1
562	1771	TTA	-1.407154846	1
224	706	TTA	-1.443515617	1
356	1144	GAT	-1.445958449	1
523	1654	CAG	-1.498023075	1
92	280	AAG	-1.498623063	1
18	52	CAG	-1.500273995	1
431	1369	GAG	-1.500273995	1
569	1792	CAG	-1.500273995	1
20	64	GAG	-1.500840688	1
99	301	CAG	-1.501056458	1
495	1570	CAG	-1.501056458	1
694	2167	GAG	-1.501056458	1
74	226	CAG	-1.502185128	1
235	739	AAG	-1.502185128	1
298	928	GAG	-1.502185128	1
187	565	GAG	-1.502475163	1
446	1414	CAG	-1.502475163	1
524	1657	AAA	-1.513833021	1
164	496	GAA	-1.566514897	1
416	1324	CAA	-1.566514897	1
463	1465	GAA	-1.566514897	1
610	1915	GAA	-1.56796345	1
352	1132	CAT	-1.614109231	1
49	151	AAC	-1.632243047	1
690	2155	GAC	-1.632243047	1
122	370	AGC	-1.63331781	1
156	472	TAC	-1.63331781	1
473	1495	GAC	-1.63331781	1
739	2302	CGG	-1.642130249	1
570	1795	AAC	-1.643606936	1
679	2122	CGG	-1.644543658	1
484	1528	TAC	-1.652003967	1

240	754	TAT	-1.652297979	1
315	979	TTC	-1.652621623	1
414	1318	CTC	-1.86648354	1
424	1348	CTG	-1.871956049	1
693	2164	CTA	-1.906757768	1
510	1615	CTG	-1.926507859	1
37	115	CTG	-1.966857443	1
681	2128	CGT	-1.967175976	1
4	10	TCT	-2	1
11	31	CTT	-2	1
75	229	TCC	-2	1
176	532	CCA	-2	1
271	847	GTT	-2	1
358	1150	GCA	-2	1
360	1156	GCT	-2	1
369	1183	GGA	-2	1
370	1186	GCT	-2	1
377	1207	GCT	-2	1
379	1213	GGG	-2	1
387	1237	GCC	-2	1
388	1240	ACA	-2	1
389	1243	CCT	-2	1
423	1345	ACT	-2	1
425	1351	CCA	-2	1
440	1396	GGG	-2	1
504	1597	GCT	-2	1
518	1639	TCT	-2	1
576	1813	GGA	-2	1
621	1948	GCT	-2	1
659	2062	CCT	-2	1
671	2098	GTT	-2	1
705	2200	CCT	-2	1
718	2239	GGA	-2	1
735	2290	GGG	-2	1
774	2407	ACC	-2	1
775	2410	TCC	-2	1
95	289	CTG	-2.08991542	1
392	1252	TTA	-2.115429835	1
623	1954	AGG	-2.290093086	1
649	2032	AGA	-2.314405279	1
279	871	ATA	-2.515103144	1
458	1450	ATA	-2.518814712	1
731	2278	CTG	-2.554649335	1
410	1306	ATA	-2.569760328	1
702	2191	CTT	-2.575769597	1
149	451	ATA	-2.585349066	1
391	1249	CAT	-2.71085924	1
203	643	TAT	-2.718821881	1
482	1522	AAT	-2.719878503	1
750	2335	AAT	-2.720771708	1
51	157	AAT	-2.721755314	1
582	1831	AGT	-2.754219154	1
492	1561	TAC	-2.844917013	1

106	322	CTC	-2.874596214	1
153	463	AGT	-2.921290001	1
53	163	ACT	-3	1
351	1129	GGG	-3	1
499	1582	GCA	-3	1
552	1741	GTA	-3	1
561	1768	CCC	-3	1
667	2086	CCT	-3	1
701	2188	ACA	-3	1
209	661	CAG	-3.030014066	1
192	580	AAT	-3.047020271	1
94	286	CAG	-3.098241605	1
409	1303	GAA	-3.125029194	1
652	2041	TTT	-3.13418299	1
441	1399	GAA	-3.135476713	1
594	1867	AGC	-3.227694355	1
31	97	AAC	-3.227849303	1
338	1048	GAC	-3.241203493	1
405	1291	GAC	-3.250037303	1
686	2143	TTC	-3.250037303	1
654	2047	TTC	-3.262787677	1
428	1360	TAC	-3.270421297	1
332	1030	TGC	-3.276643921	1
514	1627	GAC	-3.281886072	1
167	505	AGA	-3.412672406	1
353	1135	ATC	-3.711562005	1
460	1456	GGG	-4	1
475	1501	GCA	-4	1
579	1822	ACC	-4	1
257	805	GAT	-4.03408284	1
636	1993	TTT	-4.131659905	1
609	1912	AAT	-4.146882965	1
218	688	TTT	-4.149161717	1
756	2353	GAT	-4.495614034	1
180	544	GAG	-4.506889929	1
354	1138	CAG	-4.532824012	1
604	1897	TAT	-4.606387891	1
404	1288	GAA	-4.687728497	1
333	1033	CAT	-4.812533275	1
139	421	TGC	-4.87154412	1
246	772	CCA	-5	1
299	931	GCC	-5	1
583	1834	CCA	-5	1
641	2008	GTG	-5	1
131	397	TGT	-5.573671016	1
508	1609	GGC	-6	1
540	1705	GCA	-6	1
348	1120	CAT	-6.183511617	1

Supplementary Table S3. B. Detection of positions in the ACE2 sequence alignment that are likely to experience episodic positive selection (the MEME program).

**Position**	***p*-Value**
65	<0.01
214	<0.01
359	<0.01
26	0.01
93	0.01
156	0.01
429	0.01
658	0.02
740	0.02
78	0.02
343	0.02
470	0.02
657	0.02
675	0.03
782	0.04
67	0.04
206	0.05
420	0.05
